# Catalytic Antioxidant Activity of Bis-Aniline-Derived Diselenides as GPx Mimics

**DOI:** 10.3390/molecules26154446

**Published:** 2021-07-23

**Authors:** Giancarlo V. Botteselle, Welman C. Elias, Luana Bettanin, Rômulo F. S. Canto, Drielly N. O. Salin, Flavio A. R. Barbosa, Sumbal Saba, Hugo Gallardo, Gianluca Ciancaleoni, Josiel B. Domingos, Jamal Rafique, Antonio L. Braga

**Affiliations:** 1 Departamento de Química, Universidade Estadual do Centro-Oeste (UNICENTRO), Guarapuava 85040-167, PR, Brazil; 2 Departamento de Química, Universidade Federal de Santa Catarina (UFSC), Florianópolis 88040-970, SC, Brazil; welmanqmc@gmail.com (W.C.E.); luana_bettanin@hotmail.com (L.B.); driellyolek@gmail.com (D.N.O.S.); flavioauggusto@gmail.com (F.A.R.B.); hugo.gallardo@ufsc.br (H.G.); josiel.domingos@ufsc.br (J.B.D.); 3 Programa de Pós-Graduação em Ciências da Saúde, Universidade Federal de Ciências da Saúde de Porto Alegre (UFCSPA), Porto Alegre 90050-170, RS, Brazil; rfscanto@gmail.com; 4 Instituto de Química—IQ, Universidade Federal de Goiás—(UFG), Goiânia 74690-900, GO, Brazil; sumbalsaba@ufg.br; 5 Department of Chemistry and Industrial Chemistry, University of Pisa, Via G. Moruzzi 13, I-56124 Pisa, Italy; gianluca.ciancaleoni@unipi.it; 6 Instituto de Química—INQUI, Universidade Federal do Mato Grosso do Sul (UFMS), Campo Grande 79074-460, MS, Brazil; 7 Department of Chemical Sciences, Faculty of Science, University of Johannesburg, Doornfontein 2028, South Africa

**Keywords:** organoselenides, GPx, DFT, non-bonding interaction, diselenides, anilines

## Abstract

Herein, we describe a simple and efficient route to access aniline-derived diselenides and evaluate their antioxidant/GPx-mimetic properties. The diselenides were obtained in good yields via ipso-substitution/reduction from the readily available 2-nitroaromatic halides (Cl, Br, I). These diselenides present GPx-mimetic properties, showing better antioxidant activity than the standard GPx-mimetic compounds, ebselen and diphenyl diselenide. DFT analysis demonstrated that the electronic properties of the substituents determine the charge delocalization and the partial charge on selenium, which correlate with the catalytic performances. The amino group concurs in the stabilization of the selenolate intermediate through a hydrogen bond with the selenium.

## 1. Introduction

**Figure 1:**
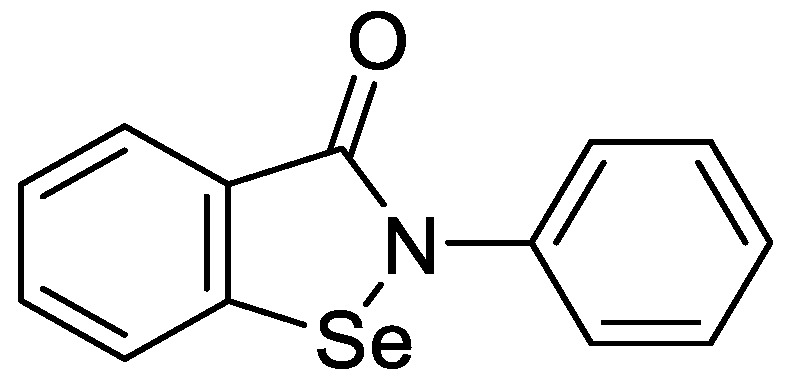
Ebselen.

In recent years, there has been an increasing interest in synthetic organoselenium compounds, mainly due to their properties as synthetic intermediates in organic transformations [1,2,3] and material sciences [4,5], as well as in medicinal chemistry [6,7,8,9]. These compounds have been recently described as good antioxidants [10,11], also presenting anti-inflammatory [12,13], antibacterial [14], antiviral [15], anticancer [16,17,18,19], anti-Alzheimer’s [20,21,22,23] and other activities [24,25,26,27,28]. Furthermore, in relation to the current pandemic of COVID-19, there are some interesting studies available that demonstrate the effectiveness of organoselenium compound (Ebselen) as an antiviral molecule, Figure 1 [29,30,31]. 

Among organoselenium compounds, diorganyl diselenides present important antioxidant and anticancer properties mainly because of the ability of these diselenides to act as mimetics of the enzyme glutathione peroxidase (GPx) [6,8,11]. This selenoenzyme possesses a residue of selenocysteine in its active site and is responsible for the reduction of peroxides to water in our organism, protecting it from oxidative stress and related diseases [32]. 

Notable among the polyfunctionalized diselenides, the presence of an amino or carbonyl group in close proximity to the selenium moiety has some unique biological features due to non-bonding interactions [33,34,35]. For example, the bis-2-aniline diselenide is reported to be a good antioxidant, preventing the oxidative stress caused by peroxynitrite and hydroperoxides [36,37,38]. Moreover, the aniline-derived diselenides, mainly with an amino group in the ortho position, give these compounds two possible reactive centers, Se-Se bond cleavage and the unshared pair of electrons on the nitrogen. This makes this class of compounds extremely flexible in functional group interconversions, making it appropriate for several transformations, mainly in the formation of selenium-containing heterocycles, such as selenamides [39], benzoselenazines [40], benzoselenazoles [41] and triazole diselenides [42].

Recently, we reported a new robust methodology for the synthesis of *o*-aniline-derived diselenides from the reduction of *o*-nitrobenzene diselenides. As part of our wider research program aimed at efficient methodologies for the synthesis of organoselenium compounds and their biological evaluation [43,44,45,46,47,48,49,50], herein, we report the application of *o*-aniline-derived diselenides as potential GPx mimics. For this purpose, different physical-chemical studies were performed to demonstrate their biological properties, i.e., kinetic profile. Furthermore, DFT studies were also carried out in order to study the electronic properties of the substituents for determining the charge delocalization on the selenium atom and its influence on catalytic performance.

## 2. Results and Discussion

**Scheme 1:**
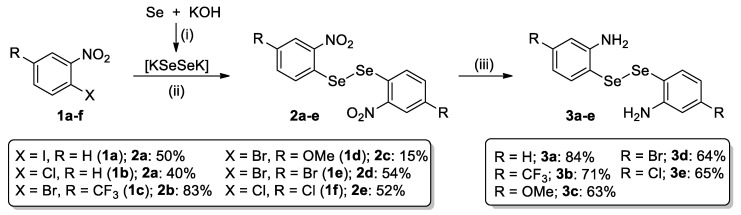
Reaction conditions: (i) Se (3.0 mmol), KOH (6.0 mmol), heated until melted for 5 min and H_2_O (6.0 mL); (ii) *o*-halonitrobenzene **1a**–**f** (1.5 mmol) and THF or DMF (1.5 mL), r.t., 2 h; (iii) *bis*-nitrobenzene diselenide **2a**–**e** (1.5 mmol) and FeSO_4_.7H_2_O (5.0 eq), methanol (25.0 mL) and H_2_O (25.0 mL), reflux, 1 h. NH_4_OH (15.0 mL), reflux, 10 min.

The bis-*o*-nitrobenzene diselenides were initially prepared through the nucleophilic aromatic substitution of *o*-halonitrobenzenes with K_2_Se_2_ (generated in situ) from a modified simple methodology [51]. After this, we carried out the reduction of bis-*o*-nitrobenzene diselenides from a well-established procedure described in the literature [52] using low-cost iron sulfate heptahydrate (FeSO_4_.7H_2_O) to synthesize the aniline-derived diselenides (Scheme 1).

**Figure 2:**
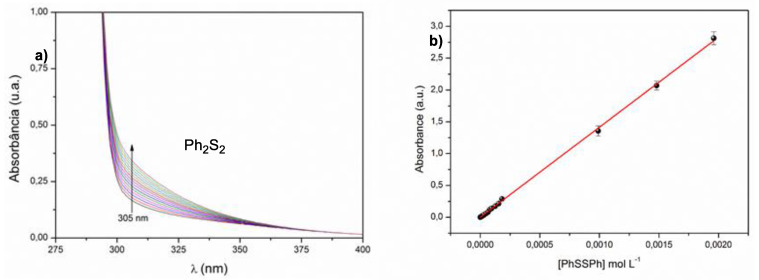
(**a**) UV-Vis spectrum of PhSH oxidation in the presence of H_2_O_2_ and diselenide **3b** as catalyst. [PhSH] = 10 mmol L^−1^, [**3b**] = 0.01 mmol L^−1^ and [H_2_O_2_] = 15 mmol L^−1^, in methanol at 25 °C; (**b**) Absorbance plotted against diphenyl disulfide concentration. The red line represents the linear fit. The coefficient of molar absorptivity in 305 nm was 1415 L mol^−1^ cm^−1^ (R^2^ = 0.9996).

The aniline-derived diselenides **3a–e** were evaluated with regard to GPx-like antioxidant activity. The catalytic parameters were obtained using the Tomoda [53] reaction model, where the synthesized diselenides were applied as catalysts in the formation of diphenyl disulfide (PhSSPh) through the reduction of hydrogen peroxide (H_2_O_2_) in the presence of thiophenol (PhSH), which is accompanied by an increase in UV/vis absorbance in 305 nm (Figure 2a). Then, the absorbance was plotted against diphenyl disulfide concentration to determine the molar absorptivity at 305 nm (Figure 2b). 

The catalytic parameters were obtained by fitting the kinetic profiles, that is, initial rate versus initial PhSH concentration, as shown in Figure 3 for diselenide **3b** (for other compounds, see Figures S31–S38, Pg. S22–S25 in Supplementary Materials), with the Michaelis–Menten equation (Equation (1)).

Table 1 shows the catalytic constant (*k_cat_*), the Michaelis–Menten constant (*K_m_*) and the catalytic efficiency (*η* where *η* = *k_cat_*/*K_m_*) for the reaction with the aniline-derived diselenides **3a** to **3e** and, for comparison, the well-known catalysts ebselen [35] and diphenyl diselenide [53]. 

**Scheme 2:**
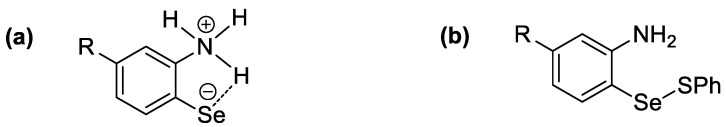
Reactive intermediates: (**a**) selenolate (a–e) and (**b**) selenyl sulfide.

The results show that the catalytic efficiency of the aniline-derived diselenides is structure-dependent, especially regarding the electronic character of the substituents at the *para* position related to selenium, with an increase in the catalytic efficiency with the electron-withdrawal capacity of the substituent (compound **3b**), once cleavage of the Se-Se bond is facilitated. These results suggest that the mechanism of these catalyzed reactions involves the formation of a zwitterionic form of the selenolate intermediate with a negative density charge in the selenium atom (Scheme 2a), which is similar to the mechanism proposed by Tomoda et al. [53].

**Figure 4:**
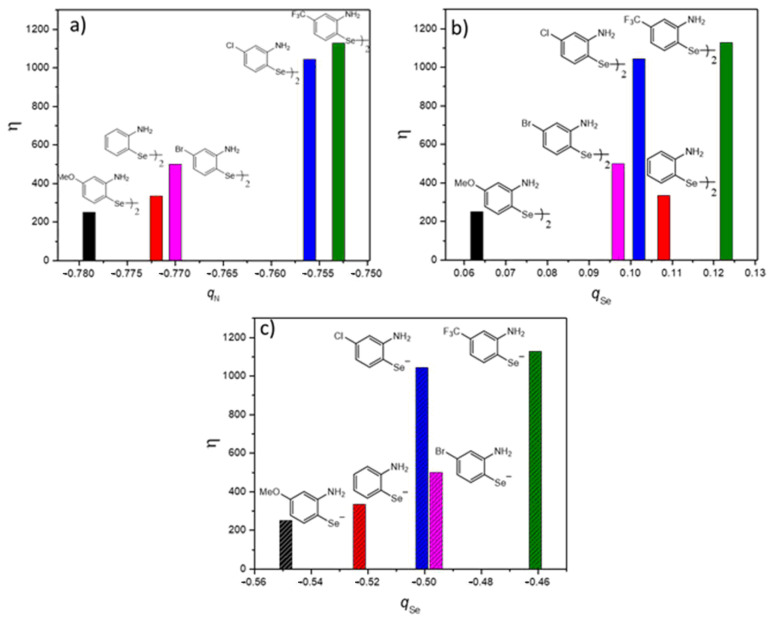
Catalytic efficiency (*η*) plotted against calculated atomic charges for (**a**) N atom (*q*_N_) of aniline-derived diselenides, (**b**) Se atom (*q*_Se_) of aniline-derived diselenides, and (**c**) Se atom (*q*_Se_) of aniline-derived selenolates.

The structures **3a**–**e** have been optimized by Density Functional Theory (DFT) at the BP86-D3/def2-TZVP level of theory, using the zero-order regular approximation (ZORA) to take the relativistic effects into account. In all the optimized geometries (except **3b**), an intramolecular hydrogen bond (HB) exists between the two amine moieties, with distances that range from 2.624 (**3d**) to 3.139 (**3a**) Å. In the case of **3b**, the electron-withdrawing -CF_3_ group likely makes the lone pair of the nitrogen less available for HBs. The electronic effect of the group in the *para* position influences all the atomic charges of the diselenide system. Indeed, the atomic charges have been computed through the Natural Population Analysis (see Computational Details) as implemented in NBO 6.0, and for the selenium, it ranges from 0.063 to 0.123 e for **3c** (the most electron-donating group) and **3b** (the most electron-withdrawing one), respectively. The atomic charge on the selenium qualitatively correlates with *η*, according to which the best catalysts have a more positive charge on the selenium and a less negative charge on the nitrogen (Figure 4 and Supplementary Materials). In addition, a similar correlation can be observed between the atomic charge of the ammonium-selenolate and *η*: in this case, the best catalysts have a less negative charge on the selenium, leading to a larger degree of charge delocalization and, consequently, a more stable intermediate. This is in agreement with the mechanism proposed by Tomoda [53]. Furthermore, the hydrogen bonding between the ammonium protons and the selenolate moiety is quite strong and stabilizes the intermediate, having an orbital interaction of 8–9 kcal/mol depending on the substituent (Supplementary Materials), hence making the catalyst more active.

Tomoda et al. [53] also proposed that another reactive intermediate is formed in the initial step from the reaction of the diselenide with PhSH, that is, the selenyl sulfide (Scheme 2b). In the case of the aniline-derived diselenides, it seems that the formation of this intermediate is destabilized by the inductive electron donor capacity of the amine groups at the ortho position, reflected in their lower catalytic efficiency when compared with the diphenyl diselenide (Table 1, entries 3 and 2, respectively).

Of the aniline-derived diselenides, the highest catalytic efficiency was observed for compound **3b** (Table 1, entry 4), which was 5 and 2 times more active than the standards ebselen and diphenyl diselenide, respectively. It is worth noting that the diselenide **3b** was more effective than ebselen, which is a pre-clinical drug candidate with pronounced biological activities, including, recently, the inhibition of protease M^pro^ from COVID-19 (SARS-CoV-2) virus [29,30,31].

**Scheme 3:**
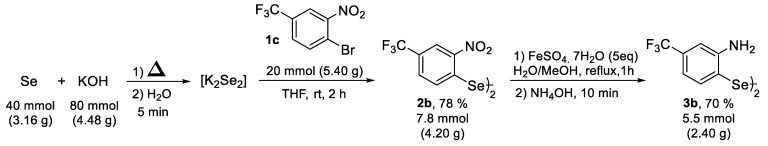
Gram-scale reaction for the synthesis of aniline-derived diselenide **3b**.

Due to the high antioxidant activity of the diselenide **3b** as a mimetic of GPx, we decided to investigate the effectiveness of this methodology at the gram scale. Thus, we performed the reaction from 20.0 mmol (5.40 g) of the o-halonitrobenzene **1c** to afford the desired nitro-diselenide **2b**, followed by its reduction to obtain the bis-aniline-derived **3b** without a significant decrease in the yields (Scheme 3), proving that this protocol could be used as a robust method in the larger-scale synthesis of this privileged structure.

## 3. Materials and Methods

### 3.1. GPx-Like Experimental Procedure

The kinetic profile of the oxidation reaction was conducted in a UV-vis Spectrophotometer, following the wavelength of diphenyl disulfide formation at 305 nm. Spectroscopic methanol was used as solvent in the oxidation reaction, and the final volume of cuvettes was kept at 2000 µL. The H_2_O_2_ and catalyst concentration were fixed in 15 × 10^−3^ mol L^−1^ and 1 × 10^−5^ mol L^−1^ respectively, and the PhSH concentration was varied from 0.5 × 10^−3^ to 15 × 10^−3^ mol L^−1^. The temperature was kept at 25 °C, and each experiment was run at least 2 times.

### 3.2. Michaelis–Menten Equation

The GPx-like kinetic profiles were treated using the Michaelis–Menten nonlinear Equation (1):

(1) $$V=\frac{k_{cat}[cat][PhSH]}{\left(K_{m}+[PhSH]\right)}$$

where the *V*_0_ was the initial velocity and *k_cat_* and *K_m_* were the catalytic rate constant and Michaelis–Menten constant, respectively. The [*cat*] and [*PhSH*] represent the concentration of the catalyst and thiophenol, respectively.

### 3.3. Computational Details

All geometries were optimized with ORCA 4.1.0, [54] using the BP86 functional in conjunction with a triple-ζ quality basis set (ZORA-TZVP) and def2/J auxiliary basis. For heavy elements (such as selenium and bromine), relativistic effects have been accounted by using the Zeroth Order Regular Approximation (ZORA) scalar correction. The dispersion corrections were introduced using the Grimme D3-parametrized correction and the Becke−Johnson damping to the DFT energy [55]. All the diselenide structures were confirmed to be local energy minima (no imaginary frequencies). Selenolate species show an unavoidable imaginary frequency correlated with the rotation of the -NH_3_ moiety. The atomic charges have been computed by the Natural Population Analysis (NPA) as implemented in NBO6 [56].

## 4. Conclusions

In conclusion, we have developed a short and robust synthetic route for the synthesis of nitro aryl and aniline-derived diselenides in good overall yields. The aniline-derived diselenides were evaluated as GPx mimetics and the diselenide **3b** substituted with the CF_3_ group showed the best results, being 5 and 2 times more effective as a GPx mimetic than the standard catalysts ebselen and diphenyl diselenide, respectively. Furthermore, DFT analysis was performed for all the diselenides, which demonstrated non-bonding interaction. This correlates with the GPx activities of these diselenides. 

## Figures and Tables

**Figure 3:**
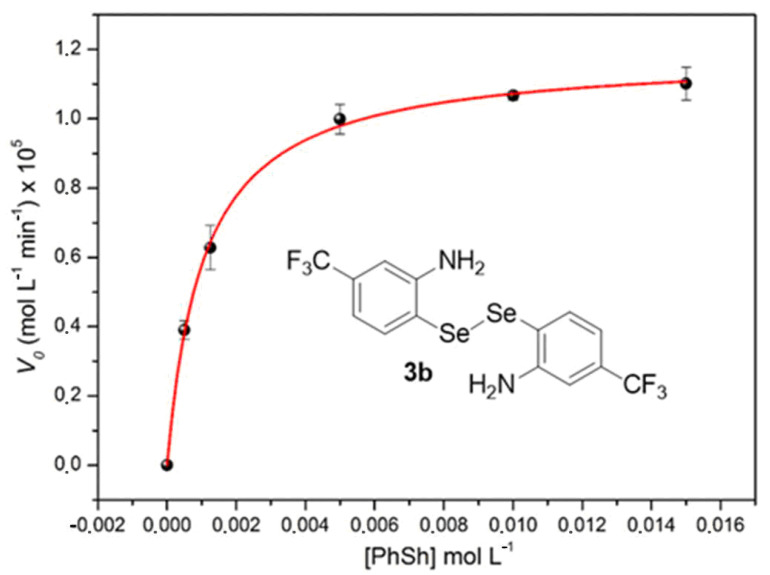
Initial rate (*V*_0_) plotted against substrate concentration. The initial rates were calculated from at least two experiments for each concentration of PhSH. The concentrations of **3b** and H_2_O_2_ were fixed at 1 × 10^−5^ and 15 × 10^−3^ mol L^−1^, respectively. The red line represents the Michaelis–Menten fit.

**Table 1:**
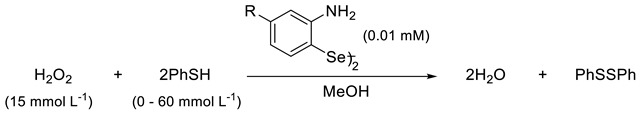
GPx-like catalytic evaluation of aniline-derived diselenides **3a**–**e**.

Entry	Catalyst (1 × 10^−5^ mol L^−1^)	*K_m_* (mol L^−1^)	*k_cat_* (min^−1^)	*η* (L mol^−1^ min^−1^)
1	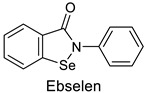	0.00170	0.422	248.65
2	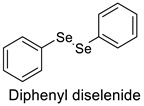	0.00114	0.601	527.78
3	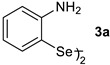	0.00134	0.446	333.40
4	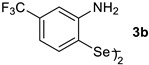	0.00105	1.185	1128.57
5	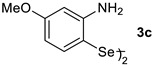	0.00187	0.470	251.51
6	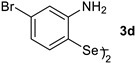	0.00081	0.405	500.11
7	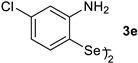	0.00088	0.918	1044.19

## Data Availability

The data presented in this study are available on request from the corresponding author.

## Supplementary Materials

The following are available online, ^1^H, and ^13^C NMR spectra of the synthesized compounds (**3a**–**e**). Figure S1: ^1^H NMR (200 MHz, CDCl_3_) Spectrum of compound **2a**. Figure S2: ^13^C NMR (50 MHz, CDCl_3_) Spectrum of compound **2a**. Figure S3: HRMS spectrum of compound **2a**. Figure S4: ^1^H NMR (200 MHz, CDCl_3_) Spectrum of compound **2b**. Figure S5: ^13^C NMR (50 MHz, CDCl_3_) Spectrum of compound **2b**. Figure S6: HRMS spectrum of compound **2b**. Figure S7: ^1^H NMR (200 MHz, CDCl_3_) Spectrum of compound **2c**. Figure S8: ^13^C NMR (50 MHz, CDCl_3_) Spectrum of compound **2c**. Figure S9: HRMS spectrum of compound **2c**. Figure S10: ^1^H NMR (200 MHz, CDCl_3_) Spectrum of compound **2d**. Figure S11: ^13^C NMR (50 MHz, CDCl_3_) Spectrum of compound **2d**. Figure S12: HRMS spectrum of compound **2d**. Figure S13: ^1^H NMR (200 MHz, CDCl_3_) Spectrum of compound **2e**. Figure S14: ^13^C NMR (50 MHz, CDCl_3_) Spectrum of compound **2e**. Figure S15: ESI-MS spectrum of compound **2e**. Figure S16:^1^H NMR (200 MHz, CDCl_3_) Spectrum of compound **3a**. Figure S17: ^13^C NMR (50 MHz, CDCl_3_) Spectrum of compound **3a**. Figure S18: ^1^H NMR (200 MHz, CDCl_3_) Spectrum of compound **3b**. Figure S19: ^13^C NMR (50 MHz, CDCl_3_) Spectrum of compound **3b**. Figure S20: HRMS spectrum of compound **3b**. Figure S21: ^1^H NMR (200 MHz, CDCl_3_) Spectrum of compound **3c**. Figure S22: ^13^C NMR (50 MHz, CDCl_3_) Spectrum of compound **3c**. Figure S23: HRMS spectrum of compound **3c.** Figure S24: ^1^H NMR (200 MHz, CDCl_3_) Spectrum of compound **3d**. Figure S25: ^13^C NMR (50 MHz, CDCl_3_) Spectrum of compound **3d**. Figure S26: HRMS spectrum of compound **3d**. Figure S27: ^1^H NMR (200 MHz, CDCl_3_) Spectrum of compound **3e**. Figure S28: ^13^C NMR (50 MHz, CDCl_3_) Spectrum of compound **3e**. Figure S29: ESI-MS spectrum of compound **3e**. Figure S30: ^77^Se NMR (76 MHz, CDCl_3_) Spectrum of compound **3b**. Figure S31: Absorbance plotted against diphenyl disulfide concentration. The red line represents the linear fit. The coefficient of molar absorptivity in 305 nm was 1415 L mol^−1^ cm^−1^ (R^2^ = 0.9996). Figure S32: Initial rate (*V*_0_) plotted against substrate concentration. The initial rates were calculated from at least two experiments for each concentration of PhSH. The concentrations of ebselen and H_2_O_2_ were fixed at 5 × 10^−5^ and 15 × 10^−3^ mol L^−1^, respectively. The red line represents the Michaelis-Menten fit. Figure S33: Initial rate (*V*_0_) plotted against substrate concentration. The initial rates were calculated from at least two experiments for each concentration of PhSH. The concentrations of diphenyl disulfide and H_2_O_2_ were fixed at 5 × 10^−5^ and 15 × 10^−3^ mol L^−1^, respectively. The red line represents the Michaelis-Menten fit. Figure S34: Initial rate (*V*_0_) plotted against substrate concentration. The initial rates were calculated from at least two experiments for each concentration of PhSH. The concentrations of **3c** and H_2_O_2_ were fixed at 5 × 10^−5^ and 15 × 10^−3^ mol L^−1^, respectively. The red line represents the Michaelis-Menten fit. Figure S35: Initial rate (*V*_0_) plotted against substrate concentration. The initial rates were calculated from at least two experiments for each concentration of PhSH. The concentrations of **3a** and H_2_O_2_ were fixed at 5 × 10^−5^ and 15 × 10^−3^ mol L^−1^, respectively. The red line represents the Michaelis-Menten fit. Figure S36: Initial rate (*V*_0_) plotted against substrate concentration. The initial rates were calculated from at least two experiments for each concentration of PhSH. The concentrations of **3b** and H_2_O_2_ were fixed at 5 × 10^−5^ and 15 × 10^−3^ mol L^−1^, respectively. The red line represents the Michaelis-Menten fit. Figure S37: Initial rate (*V*_0_) plotted against substrate concentration. The initial rates were calculated from at least two experiments for each concentration of PhSH. The concentrations of **3d** and H_2_O_2_ were fixed at 5 × 10^−5^ and 15 × 10^−3^ mol L^−1^, respectively. The red line represents the Michaelis-Menten fit. Figure S38: Initial rate (*V*_0_) plotted against substrate concentration. The initial rates were calculated from at least two experiments for each concentration of PhSH. The concentrations of **3e** and H_2_O_2_ were fixed at 5 × 10^−5^ and 15 × 10^−3^ mol L^−1^, respectively. The red line represents the Michaelis-Menten fit. Table S1: Atomic charges of nitrogen and selenium according to NPA. Table S2: Donor-acceptor second order perturbation analysis. Table S3: DFT-computed energies for optimized geometries (in kcal/mol).

## Sample Availability

Samples of the compounds can be checked with the author, G.V.B.
