# Supplementary material for: Catalytic Antioxidant Activity of Bis-Aniline-Derived Diselenides as GPx Mimics
Source: Molecules. 2021 Jul 23;26(15):4446. doi: 10.3390/molecules26154446 (PMC8347129; doi:10.3390/molecules26154446)
Supplement: Supplementary file 1 [file molecules-26-04446-s001.zip › molecules-1291350-supplementary.pdf]

Catalytic Antioxidant Activity of Bis-Aniline-Derived Diselenides as GPx Mimics

Giancarlo V. Botteselle [1,*], Welman C. Elias [2], Luana Bettanin [2], Rômulo F. S. Canto [3], Drielly N. O. Salin [2], Flavio A. R. Barbosa [2], Sumbal Saba [4], Hugo Gallardo [2], Gianluca Ciancaleoni [5], Josiel B. Domingos [2], Jamal Rafique [6,*] and Antonio L. Braga [2,7,*]

[1] Departamento de Química, Universidade Estadual do Centro-Oeste (UNICENTRO), 85040-167, Guarapuava, PR - Brazil.
[2] Departamento de Química, Universidade Federal de Santa Catarina (UFSC), 88040-970, Florianópolis, SC - Brazil.
[3] Programa de Pós-Graduação em Ciências da Saúde, Universidade Federal de Ciências da Saúde de Porto Alegre (UFCSPA), 90050-170, Porto Alegre, RS - Brazil.
[4] Instituto de Química - IQ, Universidade Federal de Goiás – (UFG), 74690-900, Goiânia - GO – Brazil.
[5] Department of Chemistry and Industrial Chemistry, University of Pisa, Via G. Moruzzi 13, I-56124, Pisa – Italy;
[6] Instituto de Química - INQUI, Universidade Federal do Mato Grosso do Sul (UFMS), 79074-460, Campo Grande, MS - Brazil.
[7] Department of Chemical Sciences, Faculty of Science, University of Johannesburg, Doornfontein, 2028, South Africa.

* Correspondence: giancarlo@unicentro.br (G.V.B); jamal.chm@gmail.com and jamal.rafique@ufms.br (J.R.); braga.antonio@ufsc.br (A.L.B.)

Table of contents:

Materials and Methods
General procedure for the synthesis of *bis-o*-nitrobenzene diselenides (2a-e)
General procedure for the synthesis of aniline-derived diselenides (3a-e)

Figure S1. ^{1}H NMR (200 MHz, $CDCl_3$) Spectrum of compound **2a**.
Figure S2. ^{13}C NMR (50 MHz, $CDCl_3$) Spectrum of compound **2a**.
Figure S3. HRMS spectrum of compound **2a**.
Figure S4. ^{1}H NMR (200 MHz, $CDCl_3$) Spectrum of compound **2b**.
Figure S5. ^{13}C NMR (50 MHz, $CDCl_3$) Spectrum of compound **2b**.
Figure S6. HRMS spectrum of compound **2b**.
Figure S7. ^{1}H NMR (200 MHz, $CDCl_3$) Spectrum of compound **2c**.
Figure S8. ^{13}C NMR (50 MHz, $CDCl_3$) Spectrum of compound **2c**.
Figure S9. HRMS spectrum of compound **2c**.
Figure S10. ^{1}H NMR (200 MHz, $CDCl_3$) Spectrum of compound **2d**.
Figure S11. ^{13}C NMR (50 MHz, $CDCl_3$) Spectrum of compound **2d**.
Figure S12. HRMS spectrum of compound **2d**.
Figure S13. ^{1}H NMR (200 MHz, $CDCl_3$) Spectrum of compound **2e**.
Figure S14. ^{13}C NMR (50 MHz, $CDCl_3$) Spectrum of compound **2e**.
Figure S15. ESI-MS spectrum of compound **2e**.
Figure S16. ^{1}H NMR (200 MHz, $CDCl_3$) Spectrum of compound **3a.**
Figure S17. ^{13}C NMR (50 MHz, $CDCl_3$) Spectrum of compound **3a**.
Figure S18. ^{1}H NMR (200 MHz, $CDCl_3$) Spectrum of compound **3b**.
Figure S19. ^{13}C NMR (50 MHz, $CDCl_3$) Spectrum of compound **3b**.
Figure S20. HRMS spectrum of compound **3b**.
Figure S21. ^{1}H NMR (200 MHz, $CDCl_3$) Spectrum of compound **3c**.
Figure S22. ^{13}C NMR (50 MHz, $CDCl_3$) Spectrum of compound **3c**.
Figure S23. HRMS spectrum of compound **3c.**
Figure S24. ^{1}H NMR (200 MHz, $CDCl_3$) Spectrum of compound **3d**.
Figure S25. ^{13}C NMR (50 MHz, $CDCl_3$) Spectrum of compound **3d**.
Figure S26. HRMS spectrum of compound **3d**.

Figure S27.^{1}H NMR (200 MHz, $CDCl_3$) Spectrum of compound **3e**.
Figure S28.^{13}C NMR (50 MHz, $CDCl_3$) Spectrum of compound **3e**.
Figure S29. ESI-MS spectrum of compound **3e**.
Figure S30. ^{77}Se NMR (76 MHz, $CDCl_3$) Spectrum of compound **3b**.

Figure S31. Absorbance plotted against diphenyl disulfide concentration. The red line represents the linear fit. The coefficient of molar absorptivity in 305 nm was 1415 L mol^{-1} cm^{-1} (R^2= 0.9996).
Figure S32. Initial rate (*V_0*) plotted against substrate concentration. The initial rates were calculated from at least two experiments for each concentration of PhSH. The concentrations of ebselen and H_2O_2 were fixed at 5 x 10^{-5} and 15 x 10^{-3} mol L^{-1}, respectively. The red line represents the Michaelis-Menten fit.
Figure S33. Initial rate (*V_0*) plotted against substrate concentration. The initial rates were calculated from at least two experiments for each concentration of PhSH. The concentrations of diphenyl disulfide and H_2O_2 were fixed at 5 x 10^{-5} and 15 x 10^{-3} mol L^{-1}, respectively. The red line represents the Michaelis-Menten fit.
Figure S34. Initial rate (*V_0*) plotted against substrate concentration. The initial rates were calculated from at least two experiments for each concentration of PhSH. The concentrations of **3c** and H_2O_2 were fixed at 5 x 10^{-5} and 15 x 10^{-3} mol L^{-1}, respectively. The red line represents the Michaelis-Menten fit.
Figure S35. Initial rate (*V_0*) plotted against substrate concentration. The initial rates were calculated from at least two experiments for each concentration of PhSH. The concentrations of **3a** and H_2O_2 were fixed at 5 x 10^{-5} and 15 x 10^{-3} mol L^{-1}, respectively. The red line represents the Michaelis-Menten fit.
Figure S36. Initial rate (*V_0*) plotted against substrate concentration. The initial rates were calculated from at least two experiments for each concentration of PhSH. The concentrations of **3b** and H_2O_2 were fixed at 5 x 10^{-5} and 15 x 10^{-3} mol L^{-1}, respectively. The red line represents the Michaelis-Menten fit.
Figure S37. Initial rate (*V_0*) plotted against substrate concentration. The initial rates were calculated from at least two experiments for each concentration of PhSH. The concentrations of **3d** and H_2O_2 were fixed at 5 x 10^{-5} and 15 x 10^{-3} mol L^{-1}, respectively. The red line represents the Michaelis-Menten fit.
Figure S38. Initial rate (*V_0*) plotted against substrate concentration. The initial rates were calculated from at least two experiments for each concentration of PhSH. The concentrations of **3e** and H_2O_2 were fixed at 5 x 10^{-5} and 15 x 10^{-3} mol L^{-1}, respectively. The red line represents the Michaelis-Menten fit.

Table S1. Atomic charges of nitrogen and selenium according to NPA.
Table S2. Donor-acceptor second order perturbation analysis.
Table S3. DFT-computed energies for optimized geometries (in kcal/mol).

Materials and Methods

Experimental

Unless specified otherwise, all starting materials, reagents and solvents were commercially available. All reactions were monitored by thin-layer chromatography on silica gel plates (GF-254) and visualized with UV light, iodine tub or stained with vanillin. All the melting points were measured on a MQ APF-301. 1H-NMR and 13C-NMR spectra were recorded on a Bruker Avance 200 NMR spectrometer with tet-ramethylsilane (TMS) as an internal reference. All chemical shifts are reported in parts per million (ppm). The kinetics of the reactions was determined on a Varian UV-visible system Spectrophotometer Carey Bio 50, equipped with a temperature con-trol bath. High-resolution exact mass measurements were performed using Atmos-pheric-pressure chemical ionization (APCI), positive mode on a mass spectrometer Bruker Daltonics micrOTOF-Q II and Electrospray ionization (ESI), positive mode on a quadrupole time-of-flight Agilent QTOF-MS spectrometer. Low resolution mass spec-tra were obtained from a Shimadzu GCMS-QP5050A apparatus equipped with a DBM-5 capillary column (30 m) using electron ionization voltage of 70 eV.

General procedure for the synthesis of *bis-o*-nitrobenzene diselenides (2a-e)

To a round-bottomed flask, provided with magnetic stirring bar, the nucleophilic selenium species (K_2Se_2) was prepared *in situ* by heating (with a thermal blower) of a mixture of elemental selenium (100 mesh, 237.0 mg, 3.0 mmol) and KOH (336.0 mg, 6.0 mmol) until melted for just 5 min in the absence of an inert atmosphere. The dark red mixture formed was then cooled to room temperature and diluted in water (6.0 mL). Next the *o*-halonitrobenzene **1a-f** (1.5 mmol) and THF or DMF (1.5 mL) were added and stirred for 2 h. The product was extracted with ethyl acetate (3 x 30.0 mL) and organic phase was dried with Na_2SO_4, filtered and the solvent was completely removed under vacuum to give the crude product. The compounds were purified by flash chromatography over silica gel using ethyl acetate/hexane as the eluent.

General procedure for the synthesis of aniline-derived diselenides (3a-e)

To the reduction of bis-*o*-nitrobenzene diselenides **2a-e** (1.5 mmol), these was mixtured with methanol (25.0 mL), $FeSO_4.7H_2O$ (6.5 mmol, 1.8 g) and H_2O (25.0 mL) into a two-necked round-bottomed flask (100 mL) equipped with a reflux condenser. The reaction mixture was refluxed for 1.5 h, and then NH_4OH (15.0 mL) was added and stirred for more 10 min under reflux. After this time, the solution was cooled to room temperature, diluted with ethyl acetate (60.0 mL) and washed with H_2O (3 × 25.0 mL). The organic phase was separated, dried over Na_2SO4 and concentrated under vacuum. The residue was purified by flash chromatography on silica gel using ethyl acetate/hexane as the eluent, obtaining the desired bis-aniline-derived diselenides **3a-e** in good yields.

Spectral Data

Bis(2-nitrophenyl) diselenide (2a)[1]:

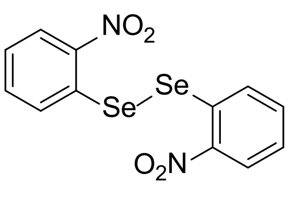

Purification by column chromatography, eluent: hexane/ethyl acetate 90:10, 50% yield (301 mg); light yellow solid, m.p.: 208-209 °C; ^{1}H NMR ($CDCl_3$, 200 MHz): δ = 8.36 (dd, J^1 = 7.8 Hz, J^2 = 1.5 Hz, 2H); 7.91 (dd, J^1 = 7.8 Hz, J^2 = 1.5 Hz, 2H); 7.56-7.39 (m, 4H); ^{13}C NMR (CDCl3, 50 MHz): δ = 146.8, 134.9, 131.8, 128.9, 127.8, 126.5; HRMS *m/z*: calc. $C_{12}H_8O_4N_2Se_2$ [M]: 403.8812; found: 403.8815; TLC (hexane/ethyl acetate 90:10): R_f = 0.25.

Bis(2-nitro-4-trifluoromethylphenyl) diselenide (2b)[2]:

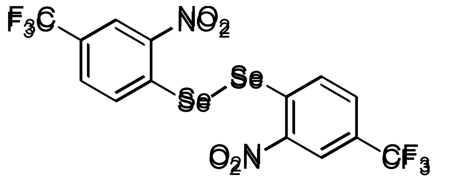

Purification by column chromatography, eluent: hexane/ethyl acetate 98:2, 83% yield for method A (671 mg) and 30 % yield for method B (81 mg), yellow solid. m.p.: 175-178 °C; ^{1}H NMR ($CDCl_3$, 200 MHz): δ = 8.65 (d, J = 1.8 Hz, 2H); 8.06 (d, J = 8.6 Hz, 2H); 7.74 (dd, J^1 = 8.6 Hz, J^2 = 1.8 Hz, 2H); ^{13}C NMR ($CDCl_3$, 50 MHz): δ = 146.5, 133.0, 132.6, 130.9 (qua, J = 34.4 Hz); 130.8 (qua, J = 3.3 Hz); 123.6 (qua, J = 3.8 Hz); 122.6 (qua, J = 271.0 Hz); HRMS *m/z*: calc. $C_{14}H_6O_4N_2Se_2F_6$ [M]: 539.8560; found: 539.8543; TLC (hexane): R_f = 0.3.

Bis(4-methoxy-2-nitrophenyl) diselenide (2c):

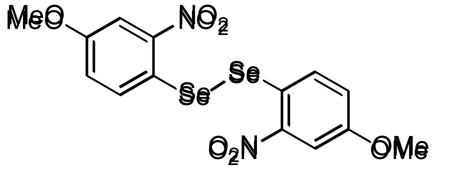

Purification by column chromatography, eluent: hexane/ethyl acetate 90:10, 15 % yield (104 mg), yellow solid, m.p.: 178-179 °C; ^{1}H NMR ($CDCl_3$, 200 MHz): δ = 7.84 (d, J = 2.8 Hz, 2H); 7.75 (d, J = 8.8 Hz, 2H); 7.09 (dd, J^1 = 8.8 Hz, J^2 = 2.8 Hz, 2H); 3.88 (s, 6H); ^{13}C NMR ($CDCl_3$, 50 MHz): δ = 159.4, 146.9, 132.4, 122.7, 119.0, 109.8, 56.0; HRMS *m/z*: calc. $C_{14}H_{12}O_6N_2Se_2$ [M]: 463.9023; found: 463.9025; TLC (hexane/ethyl acetate 80:20): R_f = 0.4.

Bis(4-bromo-2-nitrophenyl) diselenide (2d):

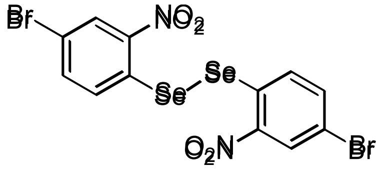

Purification by column chromatography, eluent: hexane/ethyl acetate 98:2, 54 % yield for method A (453 mg) and 90 % yield for method B (252 mg), orange solid, m.p.: 92-94 °C; ^{1}H NMR ($CDCl_3$, 200 MHz): δ = 8.49 (s, 2H); 7.75-7.57 (m, 4H); ^{13}C NMR ($CDCl_3$, 50 MHz): δ = 147.1, 137.9, 133.1, 129.3, 127.5, 121.4; HRMS *m/z*: calc. $C_{12}H_6O_4N_2Se_2Br_2$ [M]: 559.7006; found: 559.7017; TLC (hexane/ethyl acetate 90:10): R_f = 0.46.

Bis(4-chloro-2-nitrophenyl) diselenide (2e):

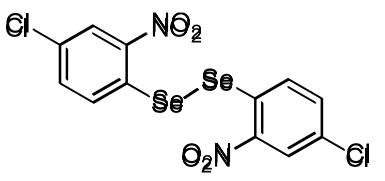

Purification by column chromatography, eluent: hexane/ethyl acetate 95:5, 52 % yield for method A (367 mg) and 81 % yield for method B (172 mg), orange solid; ^{1}H NMR ($CDCl_3$, 200 MHz): δ = 8.08 (d, J = 2.2 Hz, 2H); 7.52 (d, J = 8.6 Hz 2H); 7.18 (dd, J^1 = 8.6 Hz, J^2 = 2.2 Hz, 2H); ^{13}C NMR ($CDCl_3$, 50 MHz): δ = 146.8, 134.8, 134.1, 132.7, 126.6, 126.2; ESI-MS *m/z*: 235.9; TLC (hexane/ethyl acetate 95:5): R_f = 0.5.

Bis(2-aminophenyl) diselenide (3a)[1b,3]:

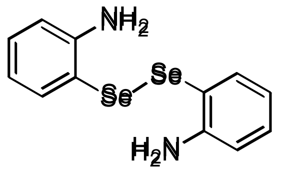

Purification by column chromatography, eluent: hexane/ethyl acetate 50:50, 84 % yield (431 mg), dark yellow solid, m.p.: 82-84 °C; ^{1}H NMR ($CDCl_3$, 200 MHz): δ = 7.35 (dd, J^1 = 7.8 Hz, J^2 = 1.4 Hz, 2H); 7.18-7.09 (m, 2H); 6.73-6.51 (m, 4H); 4.28 (br, 4H); ^{13}C NMR ($CDCl_3$, 50 MHz): δ = 149.0, 138.4, 137.5, 131.6, 118.5, 114.9; GC/MS: *m/z*(%) 346 (1), 257 (11), 255 (37), 253 (17), 252 (5), 251 (5), 184 (23), 183 (17), 181 (14), 174 (34), 173 (16), 172 (100), 170 (84), 169 (29), 168 (29), 149 (52), 83 (74), 77 (29), 65 (52), 57 (89), 55 (50), 44 (48), 43 (87); TLC (hexane/ethyl acetate 50:50): R_f = 0.75.

Bis(2-amino-4-trifluoromethlphenyl) diselenide (3b):

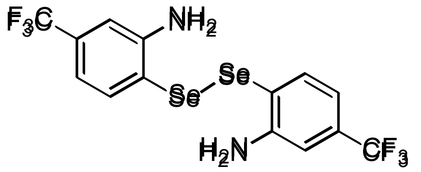

Purification by column chromatography, eluent: hexane/ethyl acetate 90:10, 71 % yield (509 mg), dark yellow solid, m.p.: 90-92 °C; ^{1}H NMR ($CDCl_3$, 200 MHz): δ = 7.42 (d, J = 8.1 Hz, 2H); 6.94 (s, 2H); 6.79 (d, J = 8.1 Hz, 2H); 4.48 (s, 4H); ^{13}C NMR ($CDCl_3$, 50 MHz): δ = 146.9, 138.6, 133.8 (qua, J = 32.2 Hz); 123.9 (qua, J = 272.6 Hz); 118.1 (qua, J = 1.5 Hz); 114.7 (qua, J = 3.8 Hz); 111.3 (qua, J = 3.8 Hz); ^{77}Se NMR (76 MHz, $CDCl_3$): δ = 401.3; HRMS *m/z*: calc. $C_{14}H_{10}N_2Se_2F_6$ [M]: 479.9076; found: 479.9079; TLC (hexane/ethyl acetate 80:20): R_f = 0.4.

Bis(4-methoxy-2-aminophenyl) diselenide (3c):

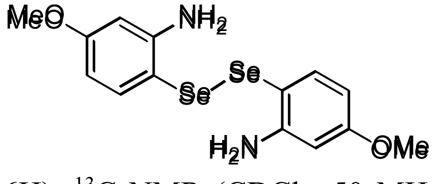

Purification by column chromatography, eluent: hexane/ethyl acetate 85:15, 63% yield (380 mg), dark orange solid; ^{1}H NMR ($CDCl_3$, 200 MHz): δ = 6.98 (d, J = 8.8 Hz, 2H); 6.03 (d, J = 2.8 Hz, 2H); 5.94 (dd, J^1 = 8.8 Hz, J^2 = 2.8 Hz 2H); 3.79 (bs, 4H); 3.45 (s, 6H); ^{13}C NMR ($CDCl_3$, 50 MHz): δ = 162.9, 150.4, 139.9, 107.0, 105.0, 99.8, 55.3; HRMS *m/z*: calc. C_7H_8ONSe: 201.9766; found: 201.9774; TLC (hexane/ethyl acetate 70:30): R_f = 0.35.

Bis(4-bromo-2-aminophenyl) diselenide (3d):

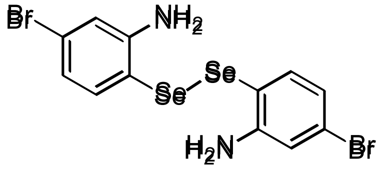

Purification by column chromatography, eluent: hexane/ ethyl acetate 98:2, 64% yield (480 mg), orange solid; m.p.: 54-55 °C; ^{1}H NMR ($CDCl_3$, 200 MHz): δ = 7.25 (d, J = 8.4 Hz, 2H); 6.91 (d, J = 2.2 Hz, 2H); 6.76 (dd, J^1 = 8.4 Hz, J^2 = 2.2 Hz, 2H); 4.15 (br, 4H); ^{13}C NMR ($CDCl_3$,

50 MHz): δ = 145.3, 133.6, 122.1, 121.7, 118.1, 107.7; HRMS *m/z*: calc. $C_{12}H_{10}N_2Se_2Br_2$ [M]: 499.7525; found: 499.7527; TLC (hexane/ethyl acetate 90:10): R_f = 0.3.

Bis(4-chloro-2-aminophenyl) diselenide (3e):

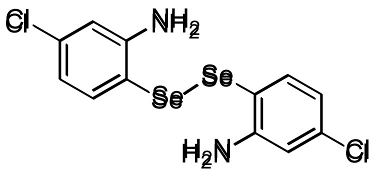

Purification by column chromatography, eluent: hexane/ethyl acetate 98:2, 65% yield (400 mg), orange solid; m.p.: 142-143; ^{1}H NMR ($CDCl_3$, 200 MHz): δ = 7.13 (d, J = 8.4 Hz, 2H); 6.73 (d, J = 2.2 Hz, 2H); 6.64 (dd, J^1 = 8.1 Hz, J^2 = 2.2 Hz, 2H); 3.98 (br, 4H); ^{13}C NMR ($CDCl_3$, 50 MHz): δ = 1438.8, 133.1, 130.2, 118.8, 117.4, 115.4; ESI-MS *m/z*: 411.1; TLC (hexane/ethyl acetate 90:10): R_f = 0.25.

5. Spectral characterization of synthesized compounds

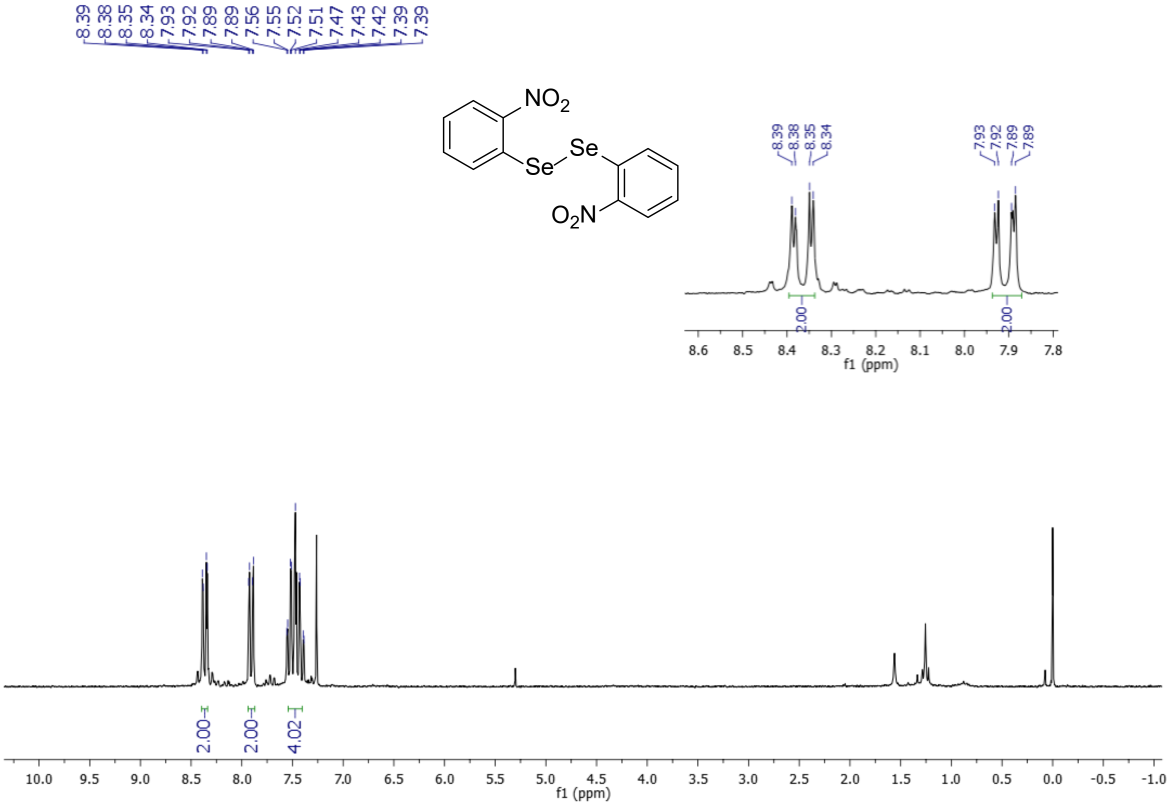

Figure S1. ^{1}H NMR (200 MHz, $CDCl_3$) Spectrum of compound **2a**.

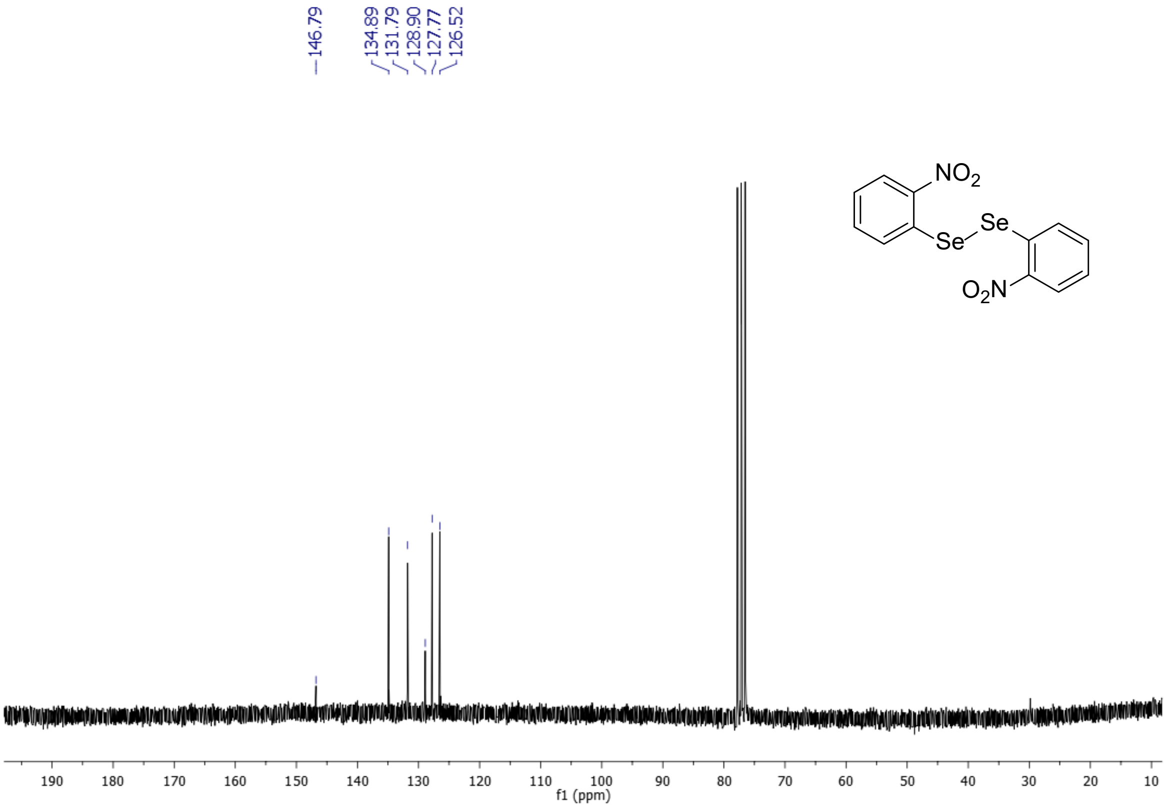

Figure S2. ^{13}C NMR (50 MHz, $CDCl_3$) Spectrum of compound **2a**.

Acquisition Parameter

Source Type	APCI	Ion Polarity	Positive	Set Nebulizer	2.5 Bar
Focus	Not active	Set Capillary	4000 V	Set Dry Heater	250 °C
Scan Begin	150 m/z	Set End Plate Offset	-500 V	Set Dry Gas	1.5 l/min
Scan End	1000 m/z	Set Collision Cell RF	150.0 Vpp	Set Divert Valve	Source

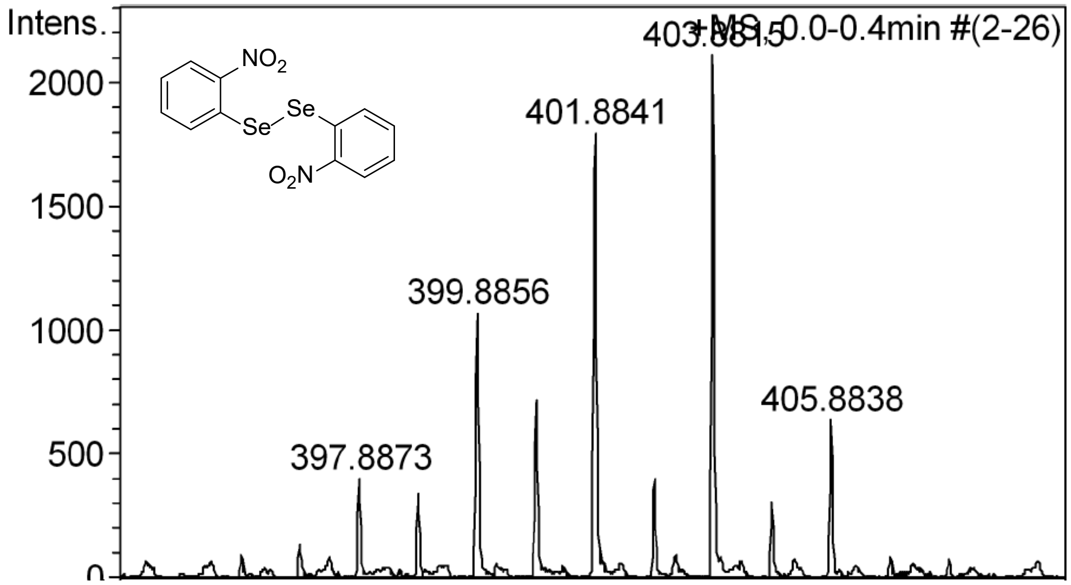

Figure S3. HRMS spectrum of compound **2a**.

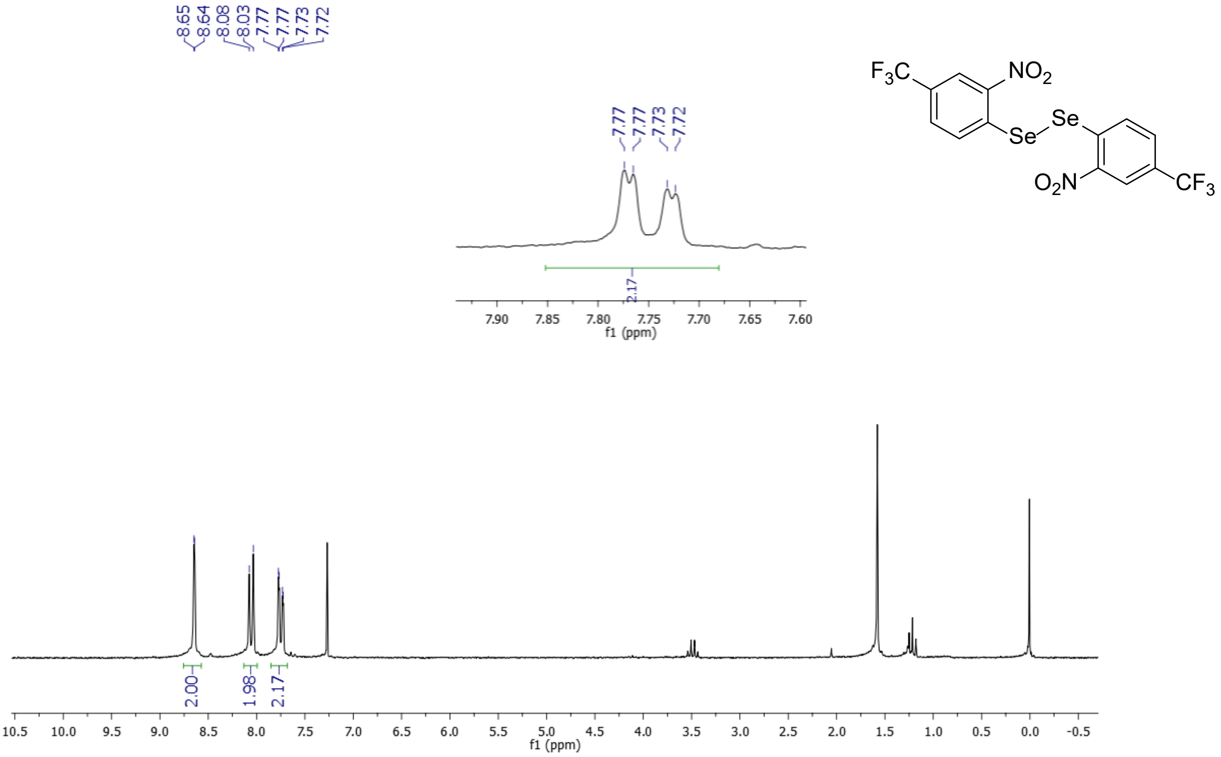

Figure S4. ^{1}H NMR (200 MHz, $CDCl_3$) Spectrum of compound **2b**.

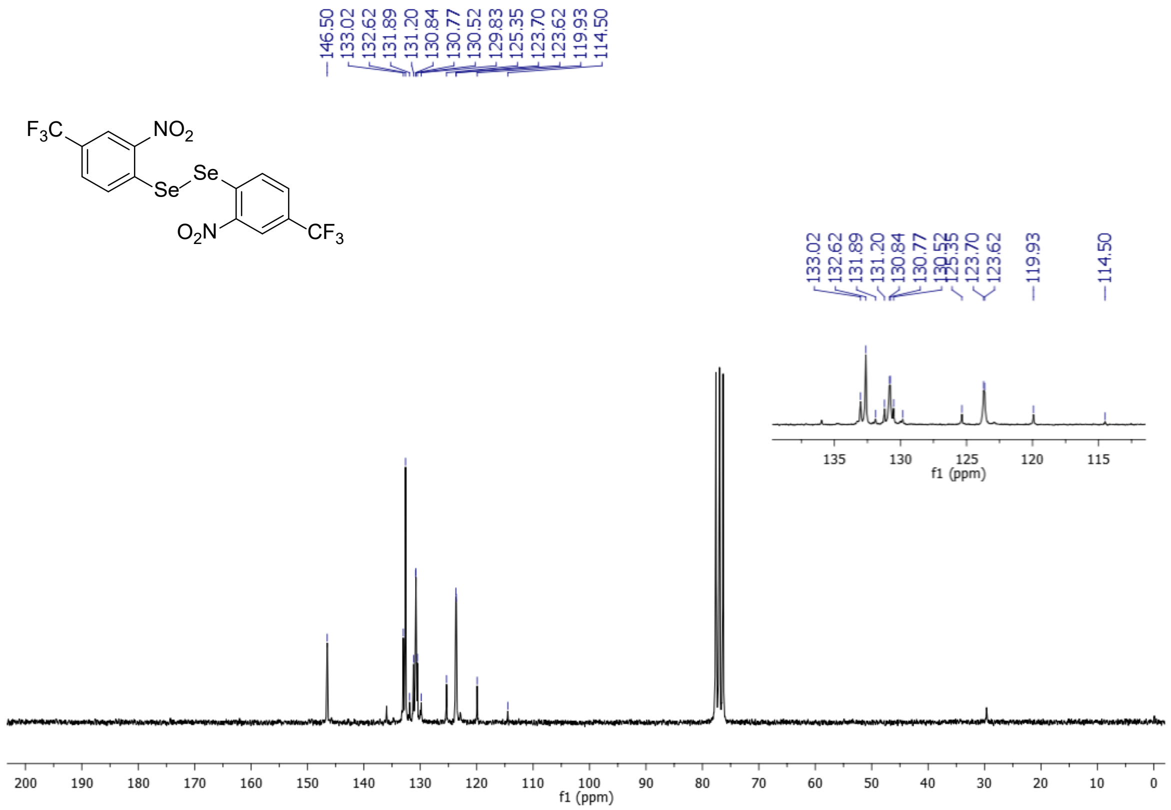

Figure S5. ^{13}C NMR (50 MHz, $CDCl_3$) Spectrum of compound **2b**.

Acquisition Parameter

Source Type	APPI	Ion Polarity	Positive	Set Nebulizer	2.0 Bar
Focus	Not active	Set Capillary	2500 V	Set Dry Heater	220 °C
Scan Begin	50 m/z	Set End Plate Offset	-500 V	Set Dry Gas	3.0 l/min
Scan End	3000 m/z	Set Collision Cell RF	600.0 Vpp	Set Divert Valve	Source

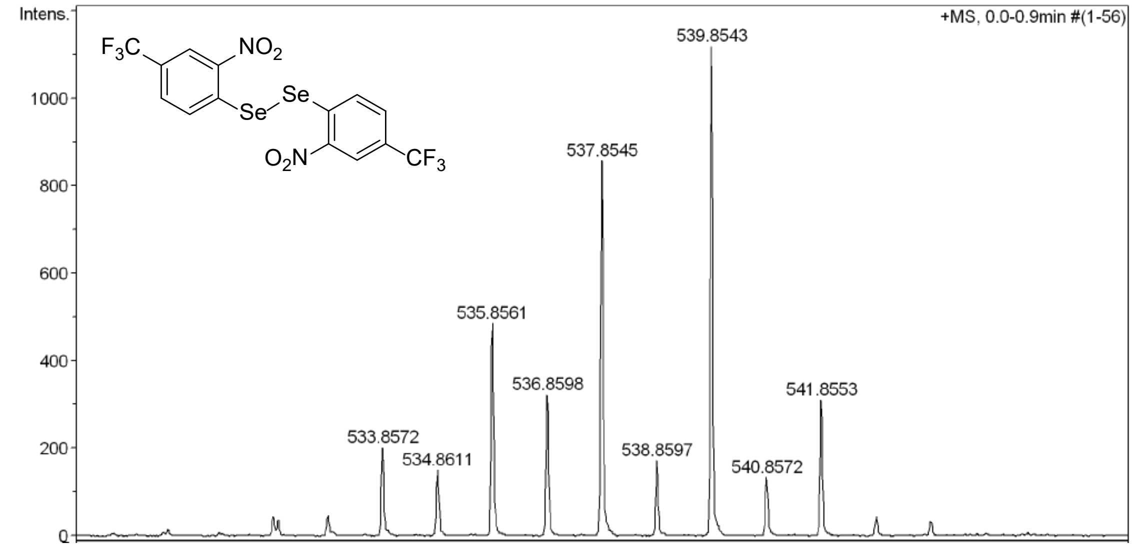

Figure S6. HRMS spectrum of compound **2b**.

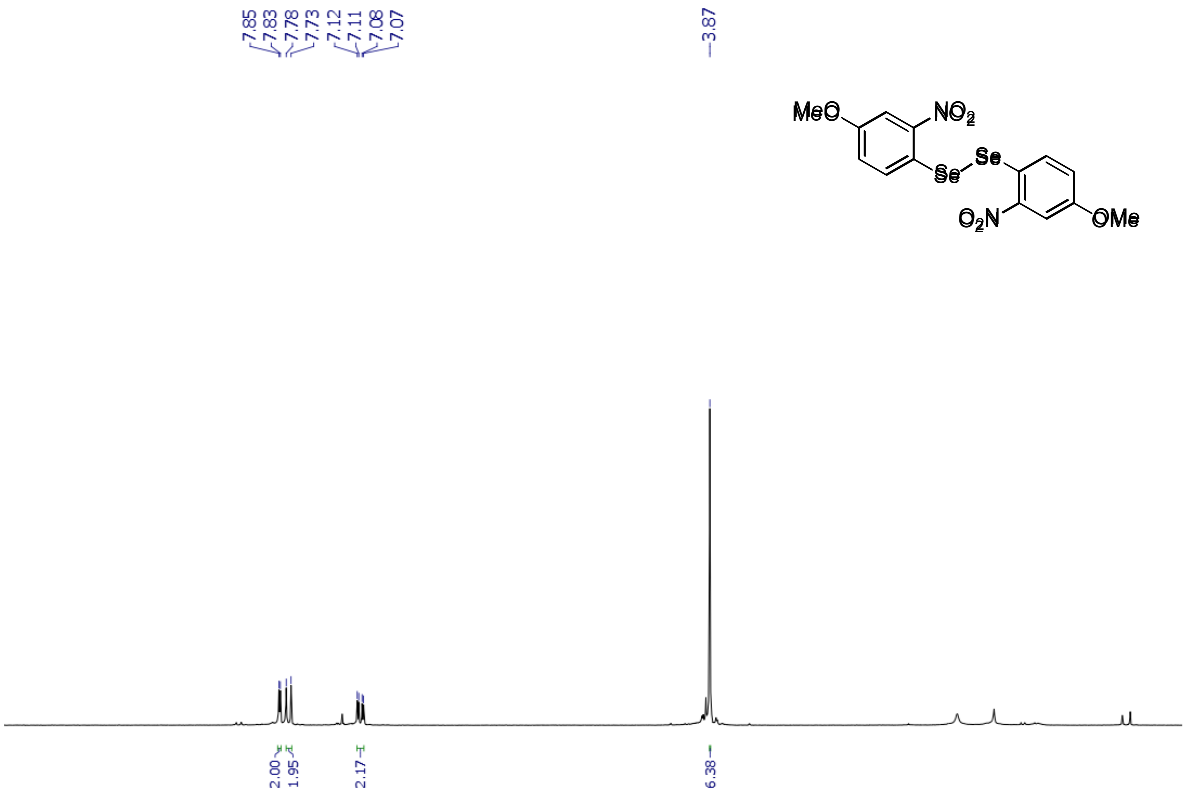

Figure S7. ^{1}H NMR (200 MHz, $CDCl_3$) Spectrum of compound **2c**.

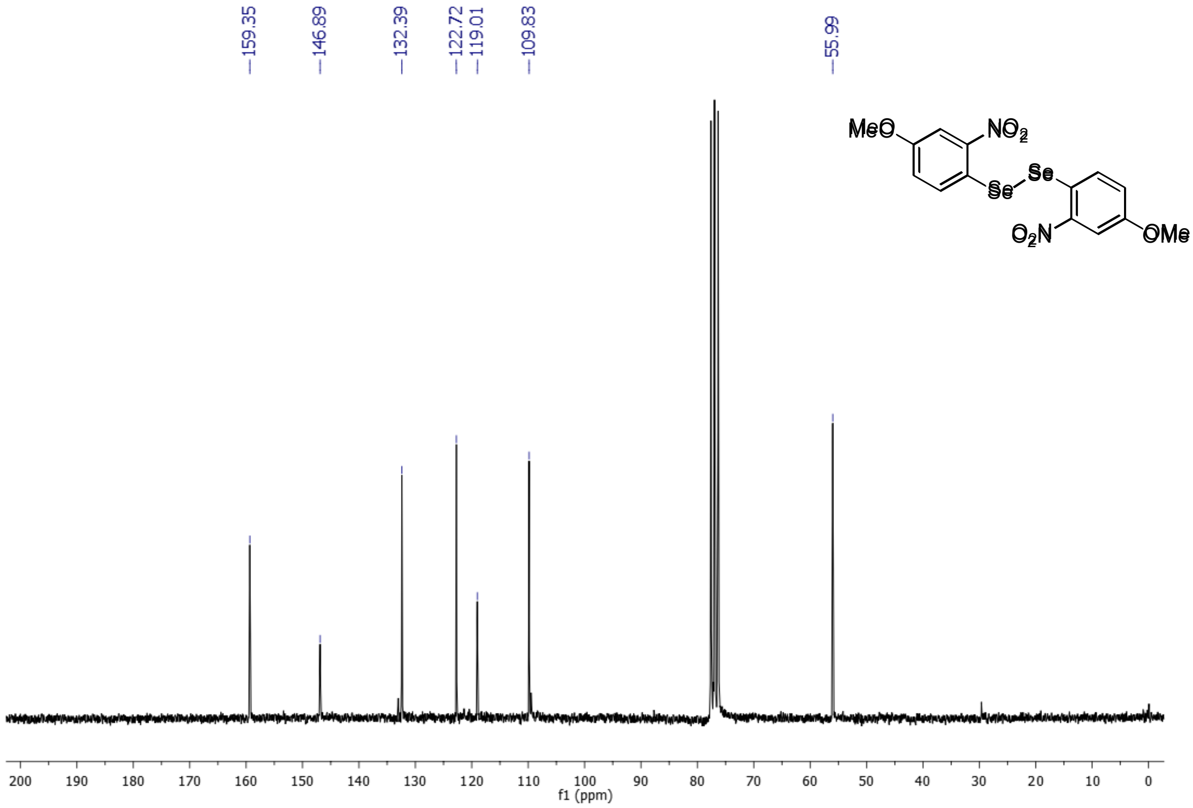

Figure S8. ^{13}C NMR (50 MHz, $CDCl_3$) Spectrum of compound **2c**.

Acquisition Parameter

Source Type	APCI	Ion Polarity	Positive	Set Nebulizer	2.5 Bar
Focus	Not active	Set Capillary	4000 V	Set Dry Heater	250 °C
Scan Begin	150 m/z	Set End Plate Offset	-500 V	Set Dry Gas	1.5 l/min
Scan End	1000 m/z	Set Collision Cell RF	200.0 Vpp	Set Divert Valve	Source

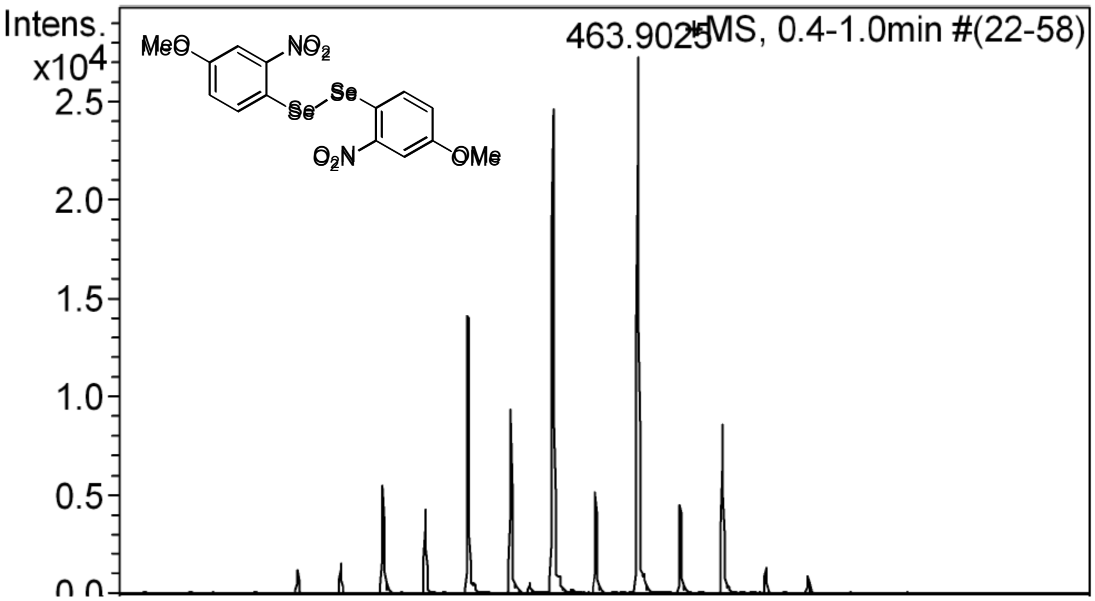

Figure S9. HRMS spectrum of compound **2c**.

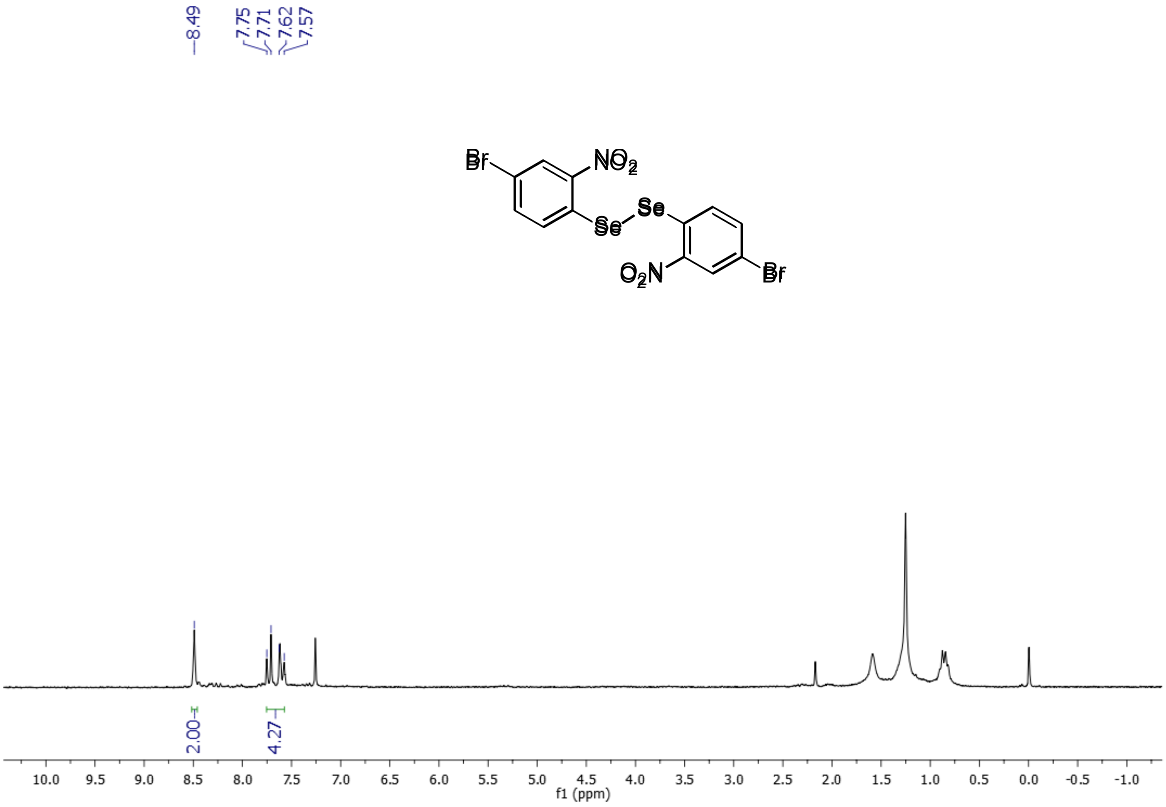

Figure S10. ^{1}H NMR (200 MHz, $CDCl_3$) Spectrum of compound **2d**.

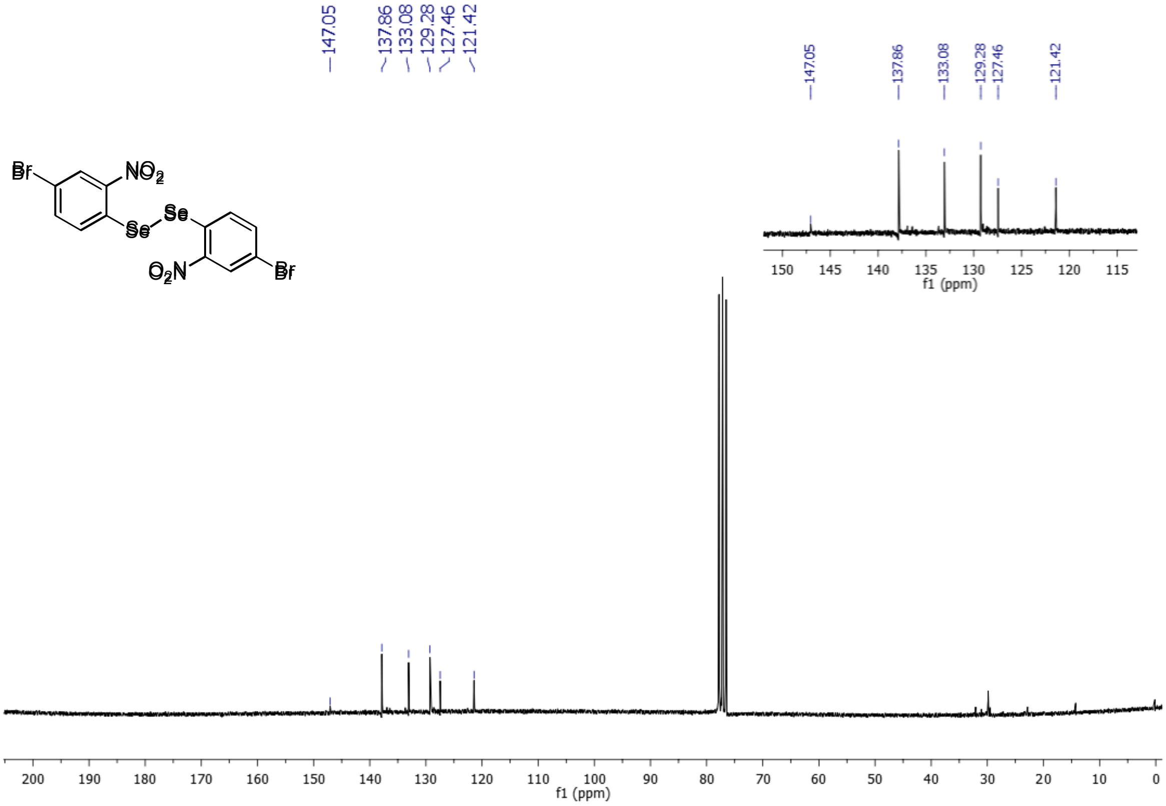

Figure S11. ^{13}C NMR (50 MHz, $CDCl_3$) Spectrum of compound **2d**.

Acquisition Parameter

Source Type	APCI	Ion Polarity	Positive	Set Nebulizer	2.5 Bar
Focus	Active	Set Capillary	4000 V	Set Dry Heater	250 °C
Scan Begin	50 m/z	Set End Plate Offset	-500 V	Set Dry Gas	1.5 l/min
Scan End	1000 m/z	Set Collision Cell RF	400.0 Vpp	Set Divert Valve	Source

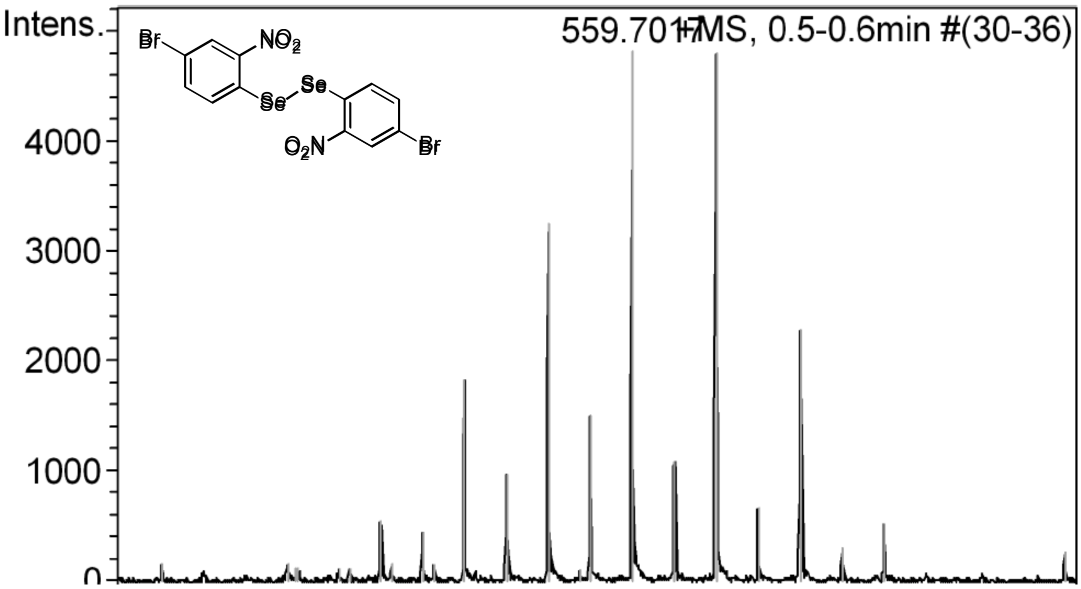

Figure S12. HRMS spectrum of compound **2d**.

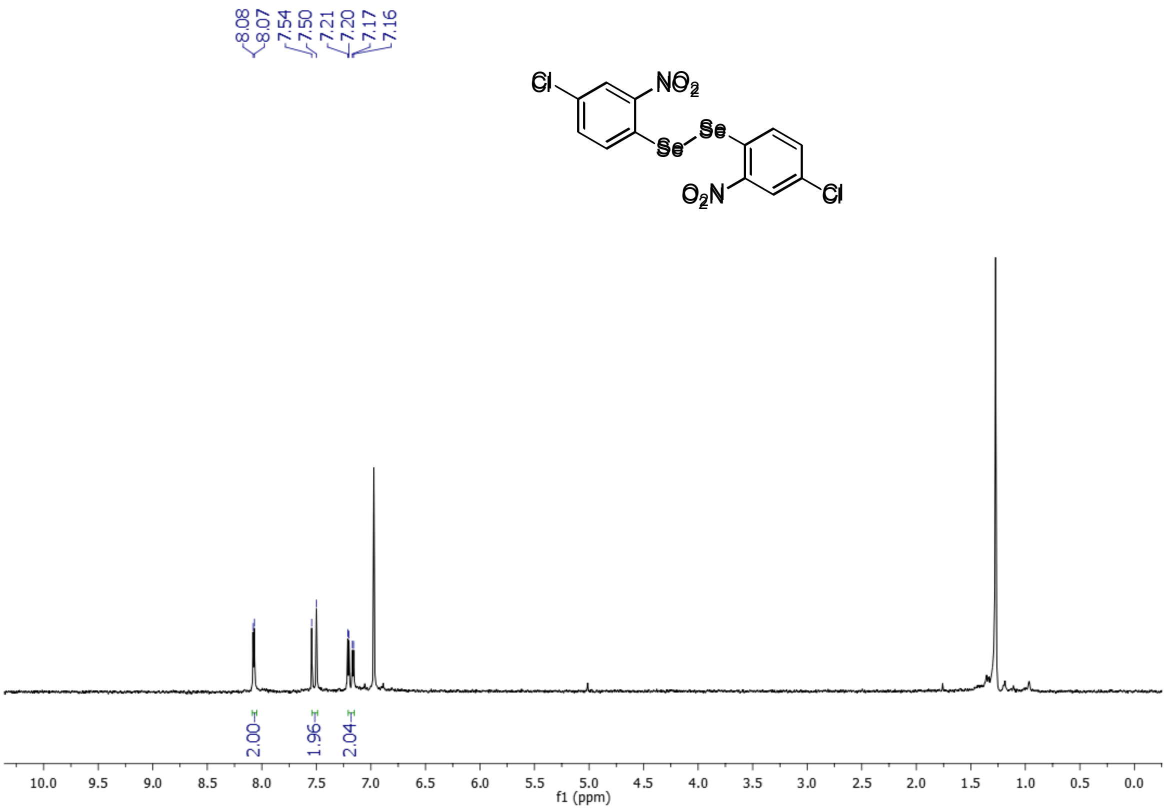

Figure S13. ^{1}H NMR (200 MHz, $CDCl_3$) Spectrum of compound **2e**.

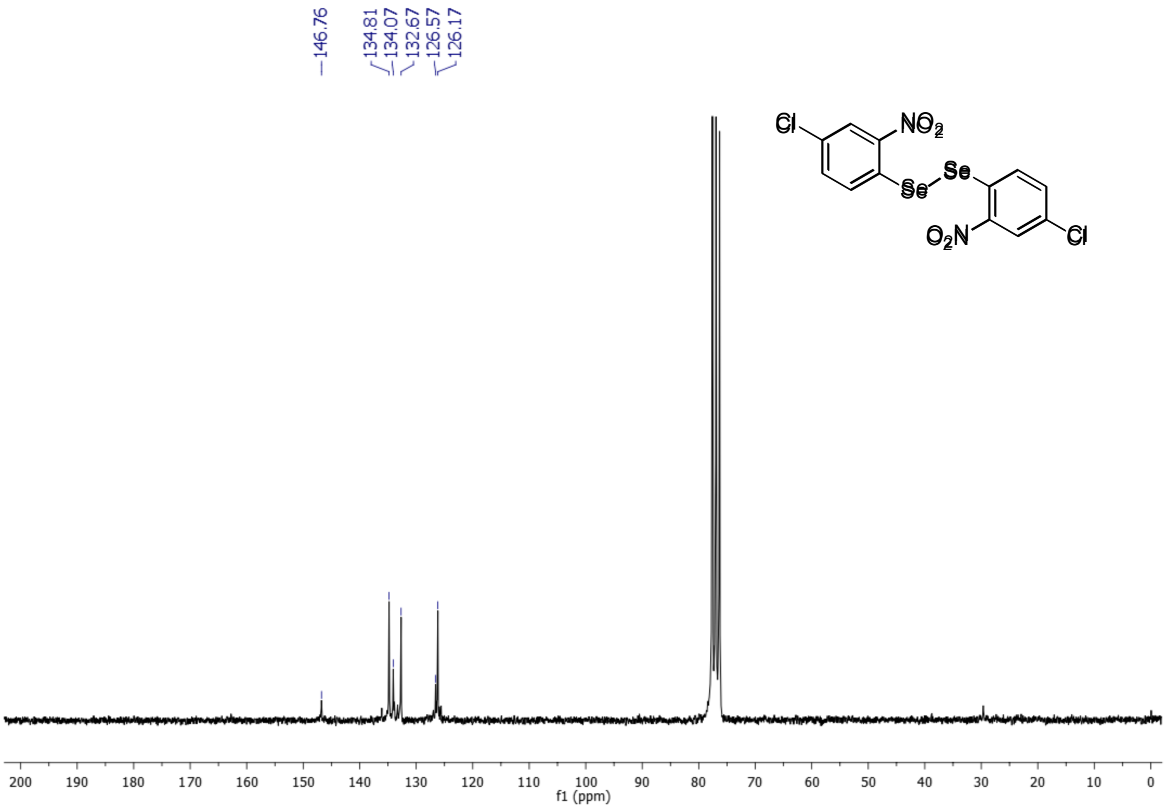

Figure S14. ^{13}C NMR (50 MHz, $CDCl_3$) Spectrum of compound **2e**.

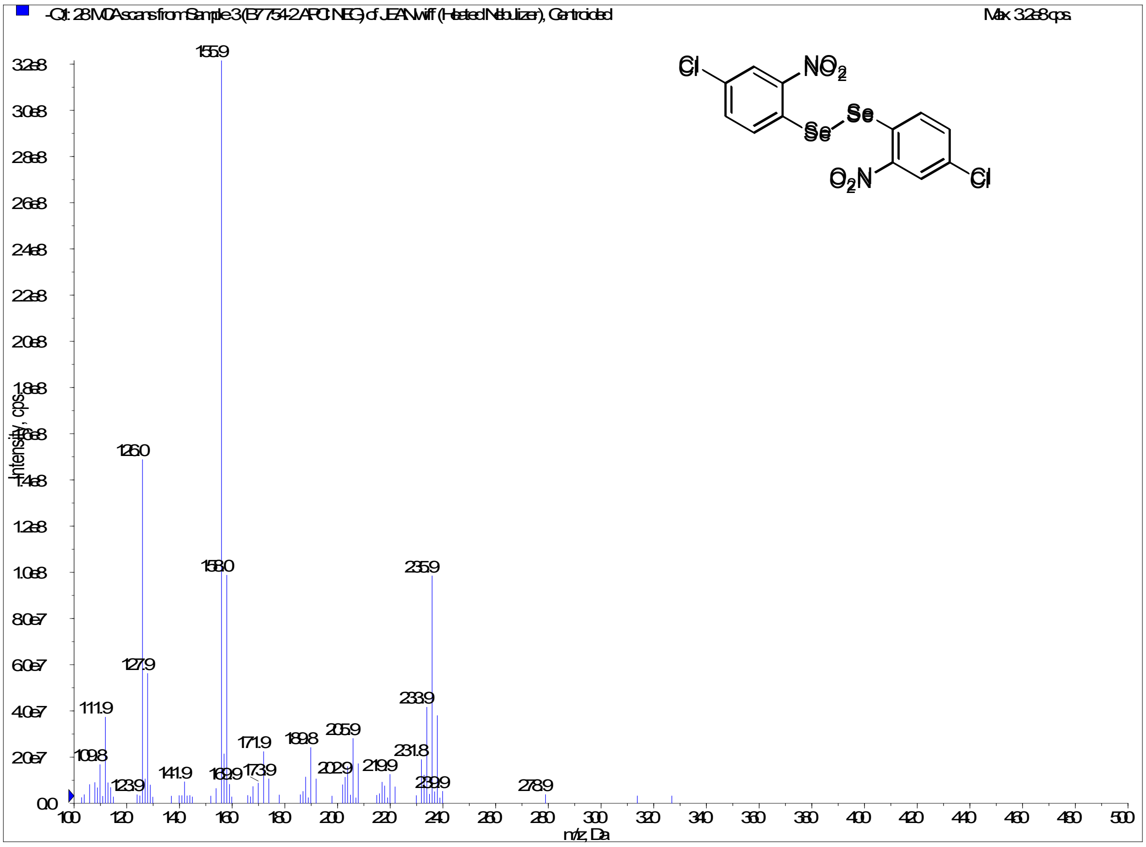

Figure S15. ESI-MS spectrum of compound **2e**.

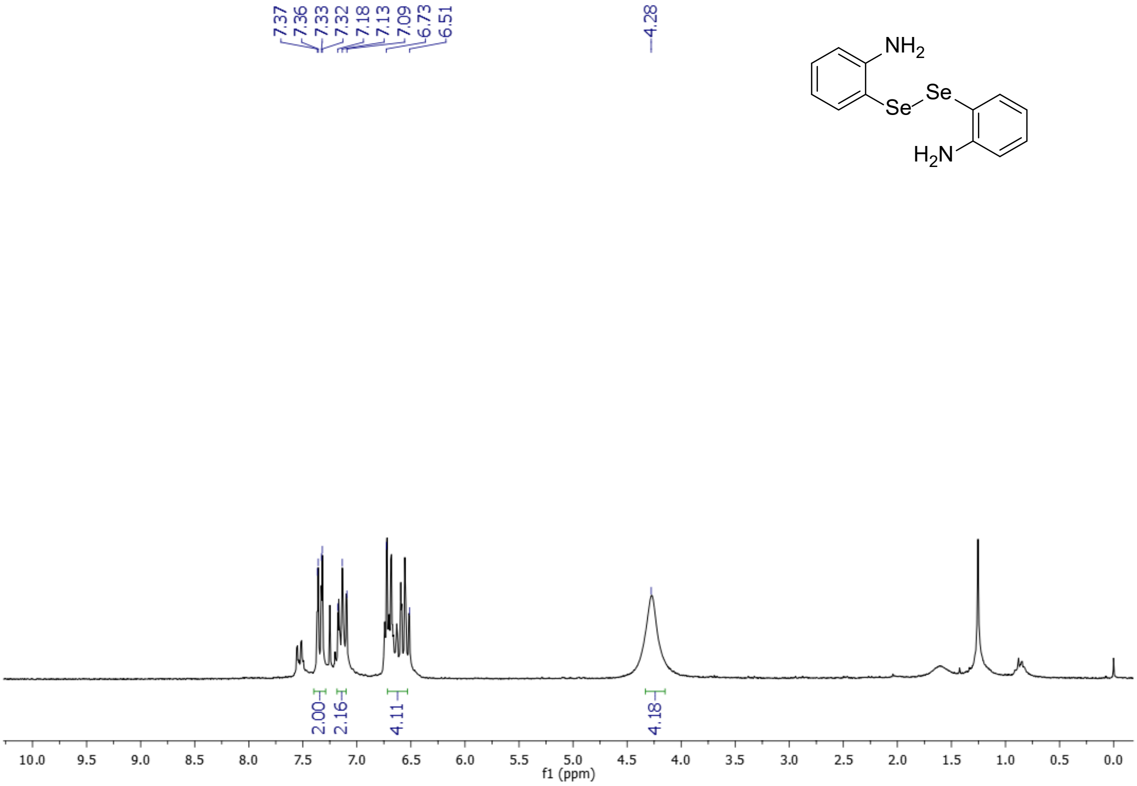

Figure S16.^{1}H NMR (200 MHz, $CDCl_3$) Spectrum of compound **3a.**

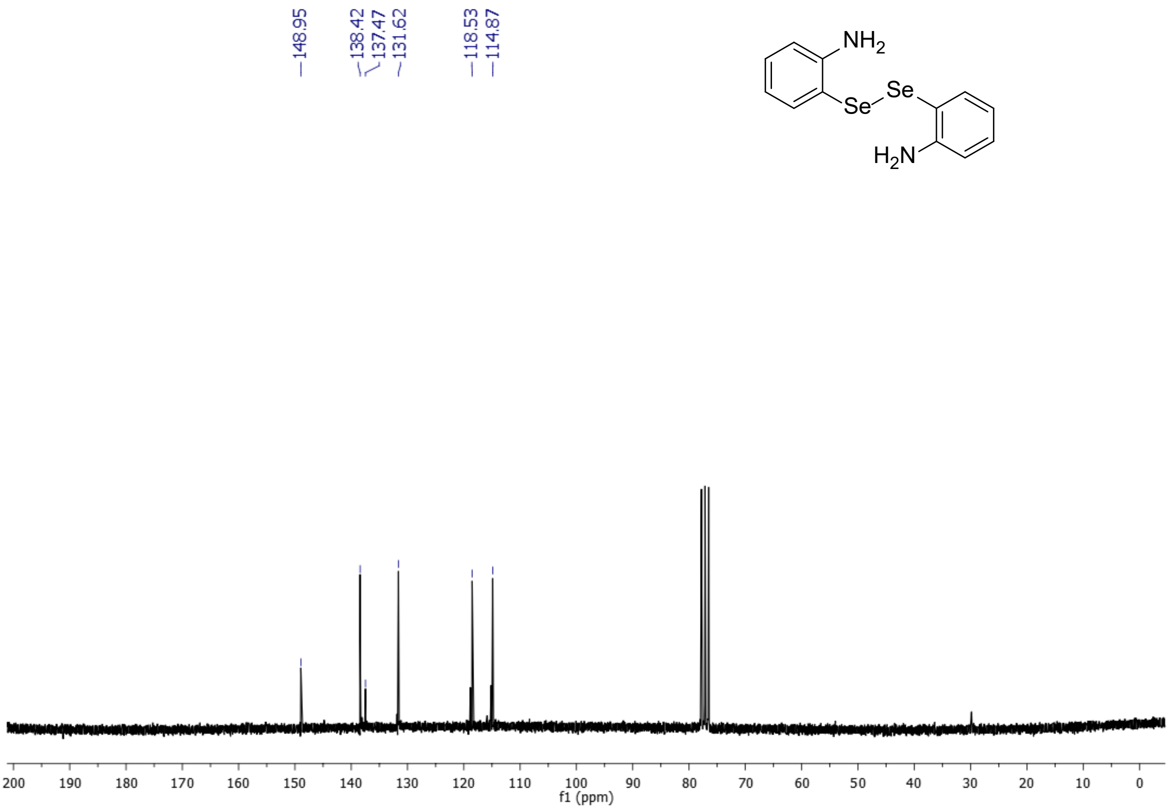

Figure S17.^{13}C NMR (50 MHz, CDCl3) Spectrum of compound **3a**.

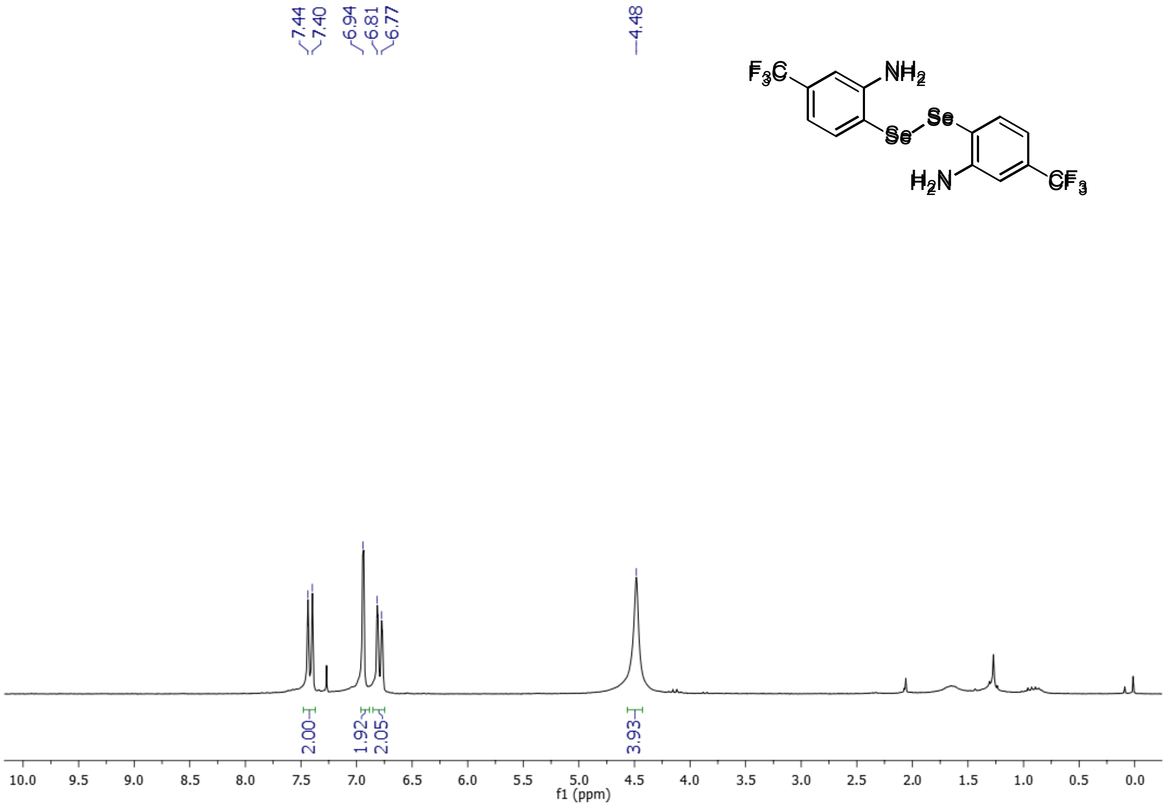

Figure S18. ^{1}H NMR (200 MHz, CDCl3) Spectrum of compound **3b**.

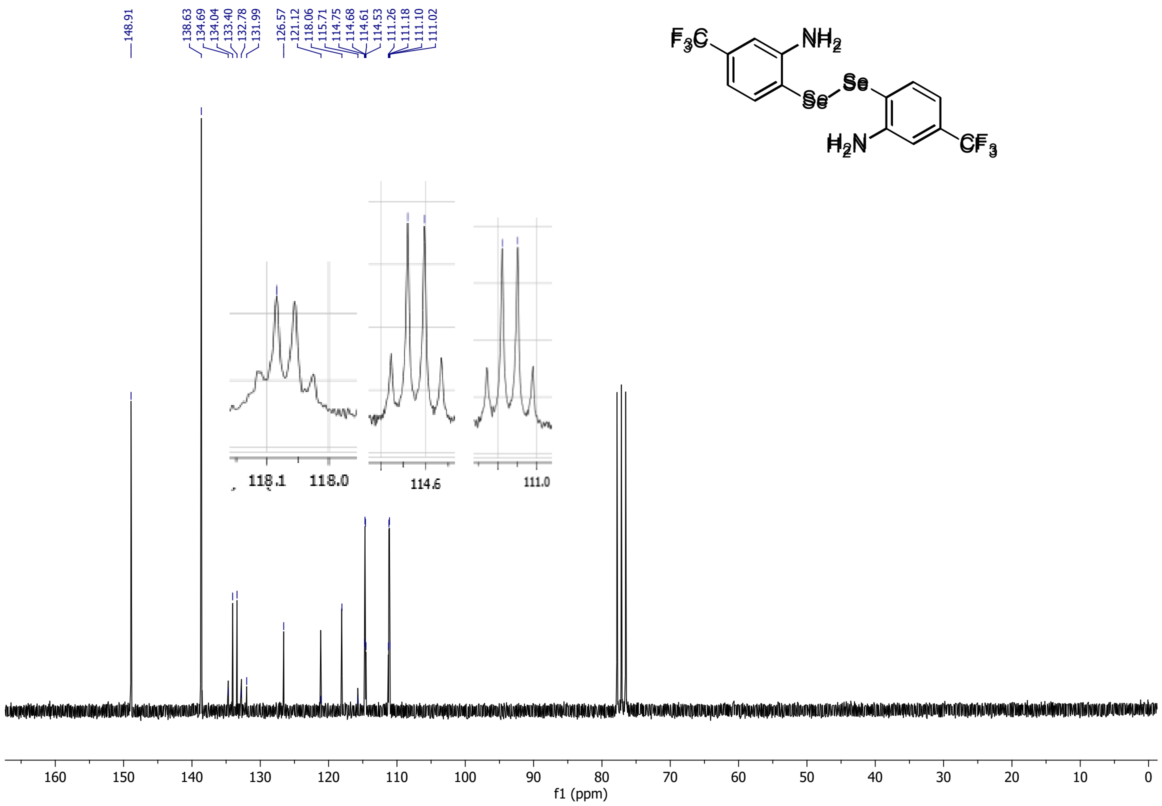

Figure S19. ^{13}C NMR (50 MHz, $CDCl_3$) Spectrum of compound **3b**.

Acquisition Parameter

Source Type	APPI	Ion Polarity	Positive	Set Nebulizer	2.5 Bar
Focus	Not active	Set Capillary	2500 V	Set Dry Heater	200 °C
Scan Begin	50 m/z	Set End Plate Offset	-500 V	Set Dry Gas	6.0 l/min
Scan End	3000 m/z	Set Collision Cell RF	600.0 Vpp	Set Divert Valve	Source

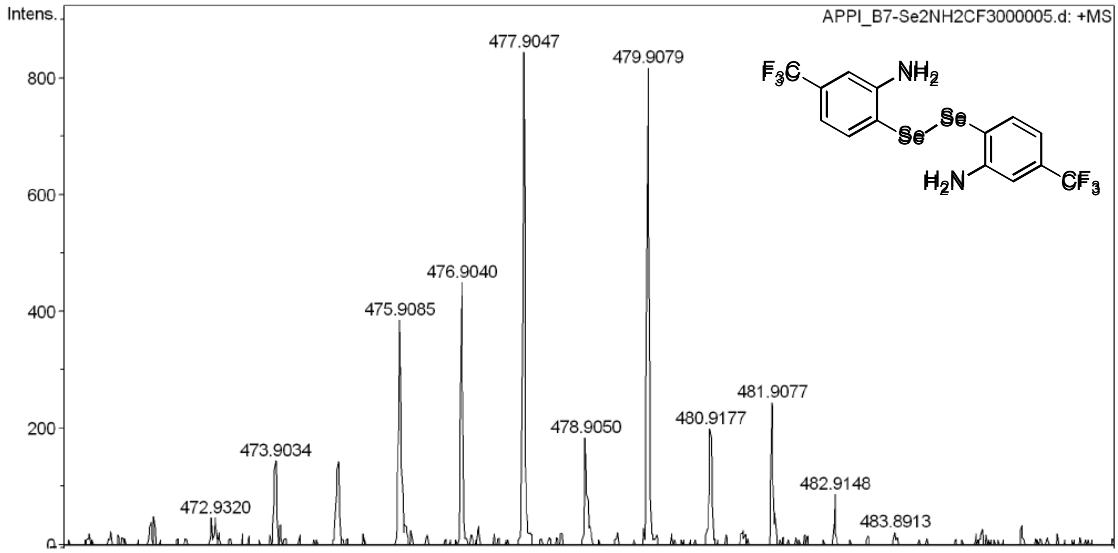

Figure S20. HRMS spectrum of compound **3b**.

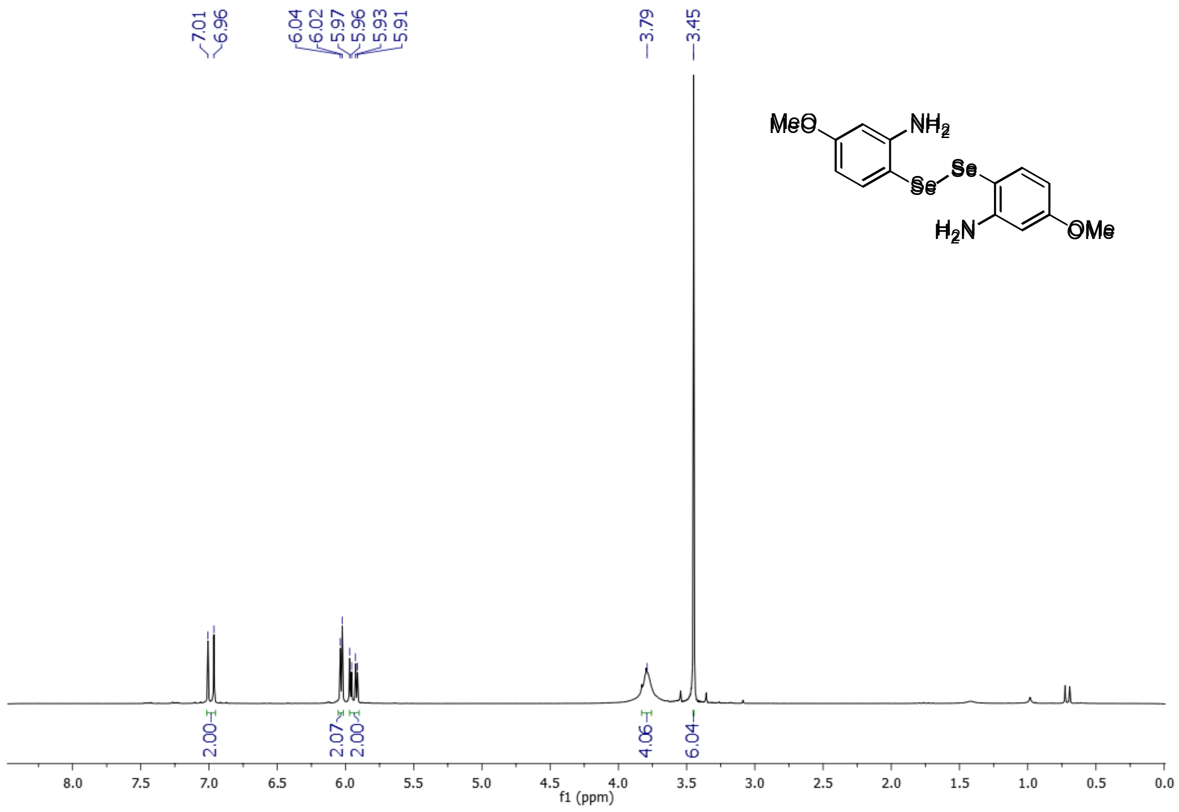

Figure S21. ^{1}H NMR (200 MHz, $CDCl_3$) Spectrum of compound **3c**.

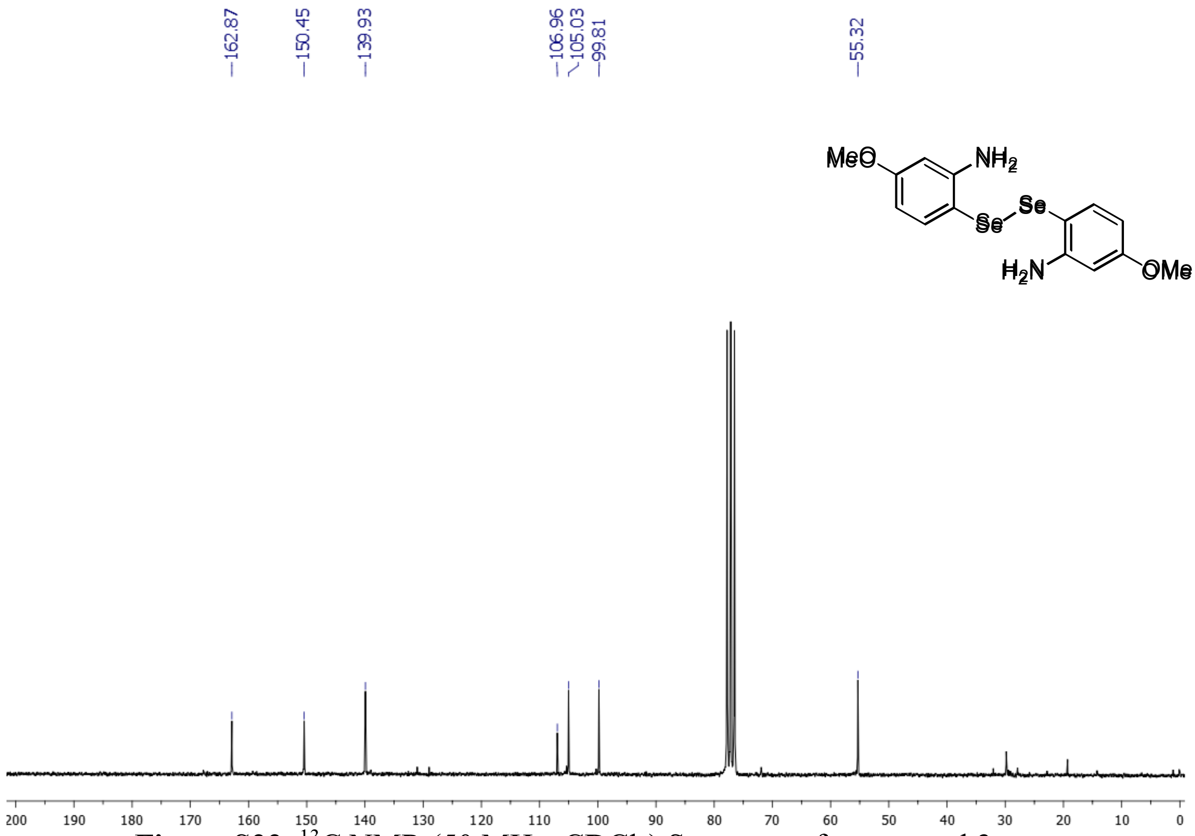

Figure S22. ^{13}C NMR (50 MHz, $CDCl_3$) Spectrum of compound **3c**.

Acquisition Parameter

Source Type	APPI	Ion Polarity	Positive	Set Nebulizer	3.0 Bar
Focus	Not active	Set Capillary	1000 V	Set Dry Heater	180 °C
Scan Begin	50 m/z	Set End Plate Offset	-500 V	Set Dry Gas	3.0 l/min
Scan End	1000 m/z	Set Collision Cell RF	100.0 Vpp	Set Divert Valve	Source

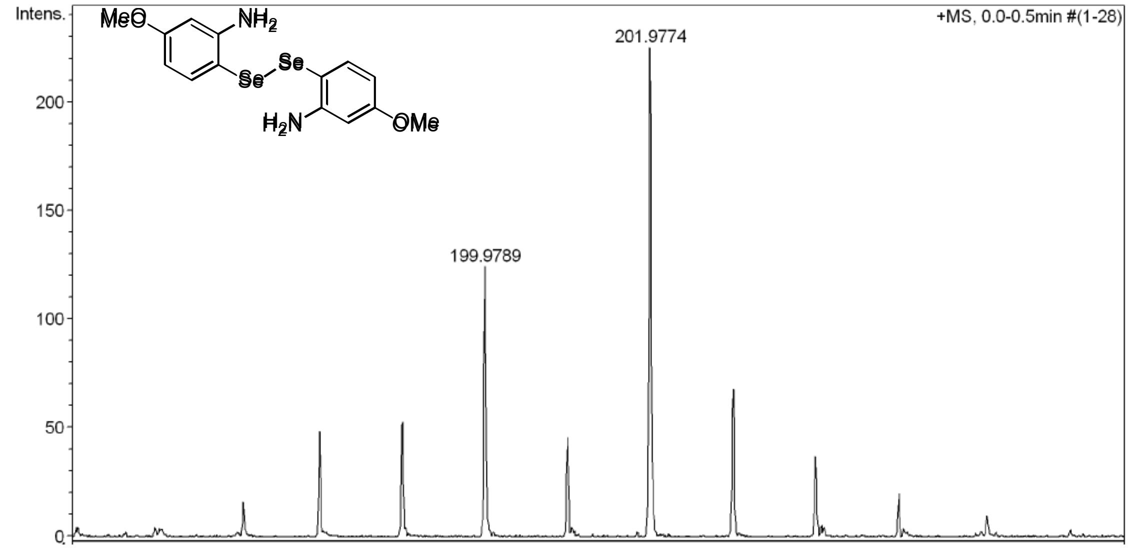

Figure S23. HRMS spectrum of compound **3c.**

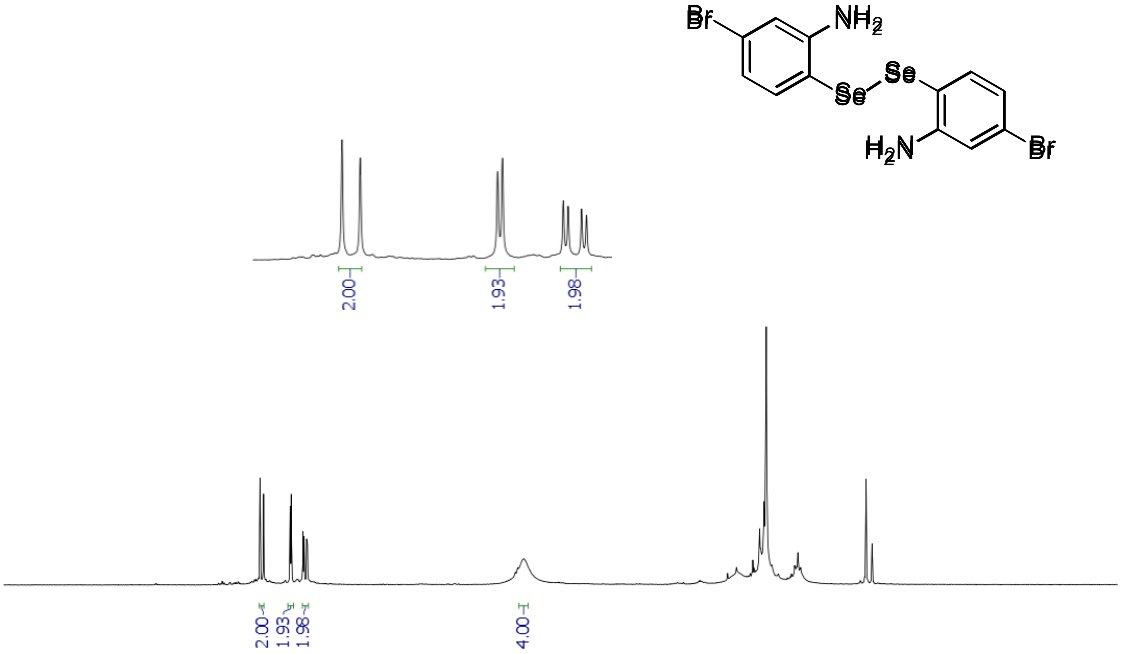

Figure S24. ^{1}H NMR (200 MHz, $CDCl_3$) Spectrum of compound **3d**.

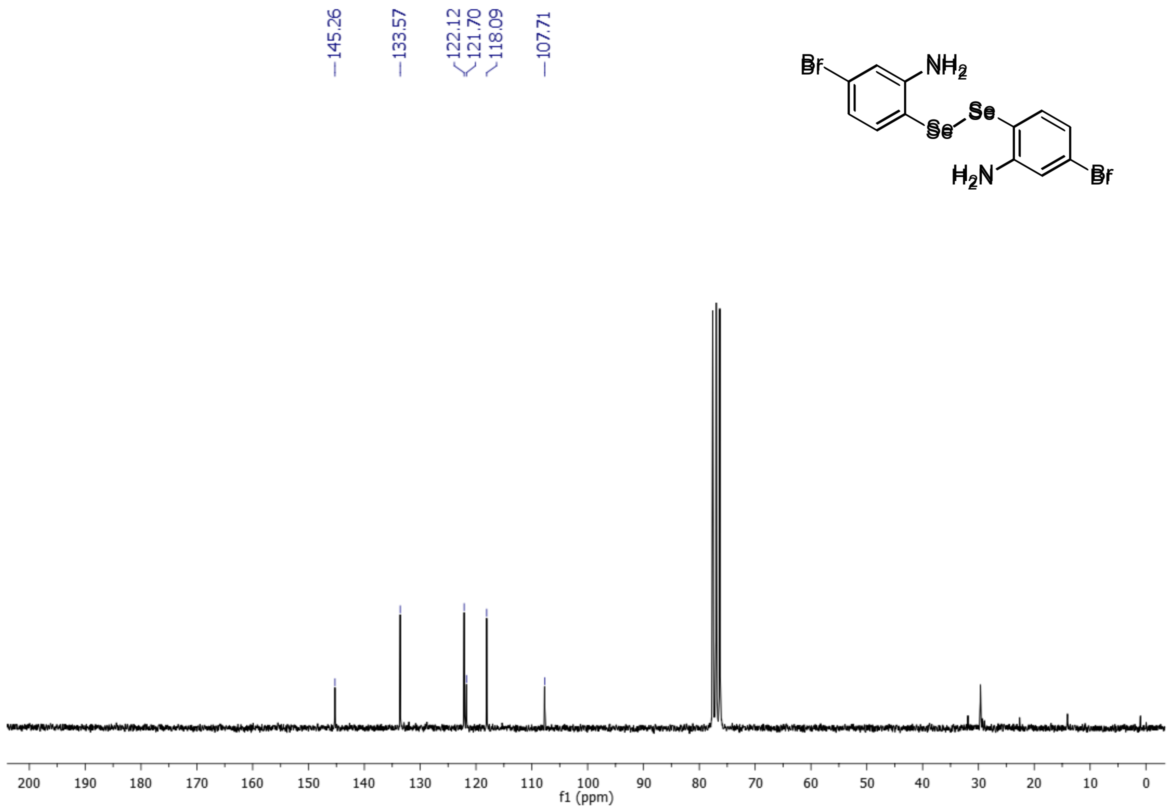

Figure S25. ^{13}C NMR (50 MHz, $CDCl_3$) Spectrum of compound **3d**.

Acquisition Parameter

Source Type	APCI	Ion Polarity	Positive	Set Nebulizer	2.5 Bar
Focus	Active	Set Capillary	4000 V	Set Dry Heater	250 °C
Scan Begin	150 m/z	Set End Plate Offset	-500 V	Set Dry Gas	1.5 l/min
Scan End	1000 m/z	Set Collision Cell RF	150.0 Vpp	Set Divert Valve	Source

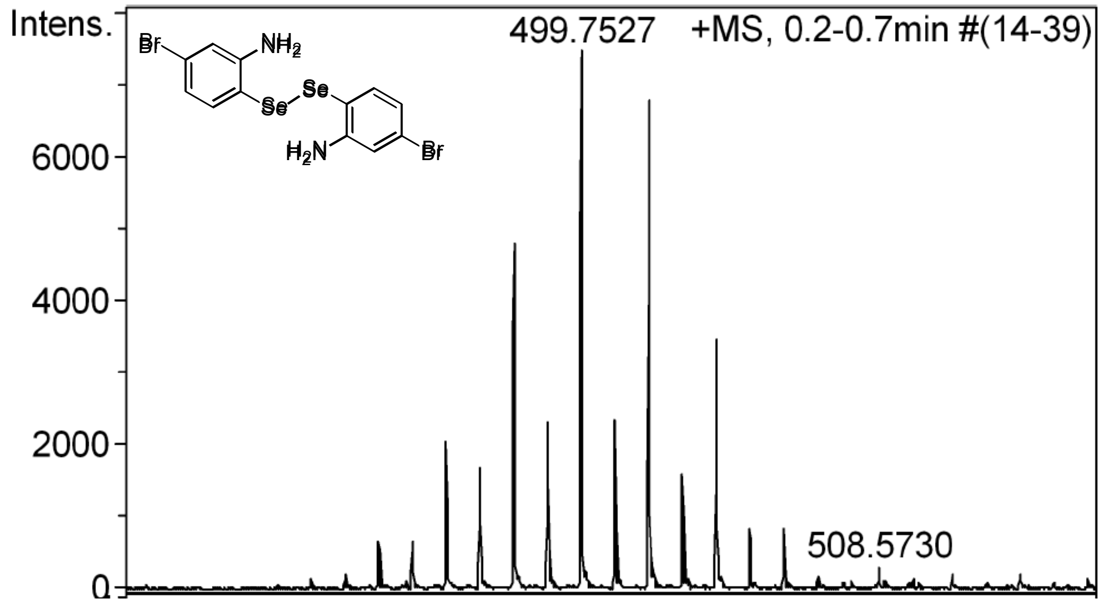

Figure S26. HRMS spectrum of compound **3d**.

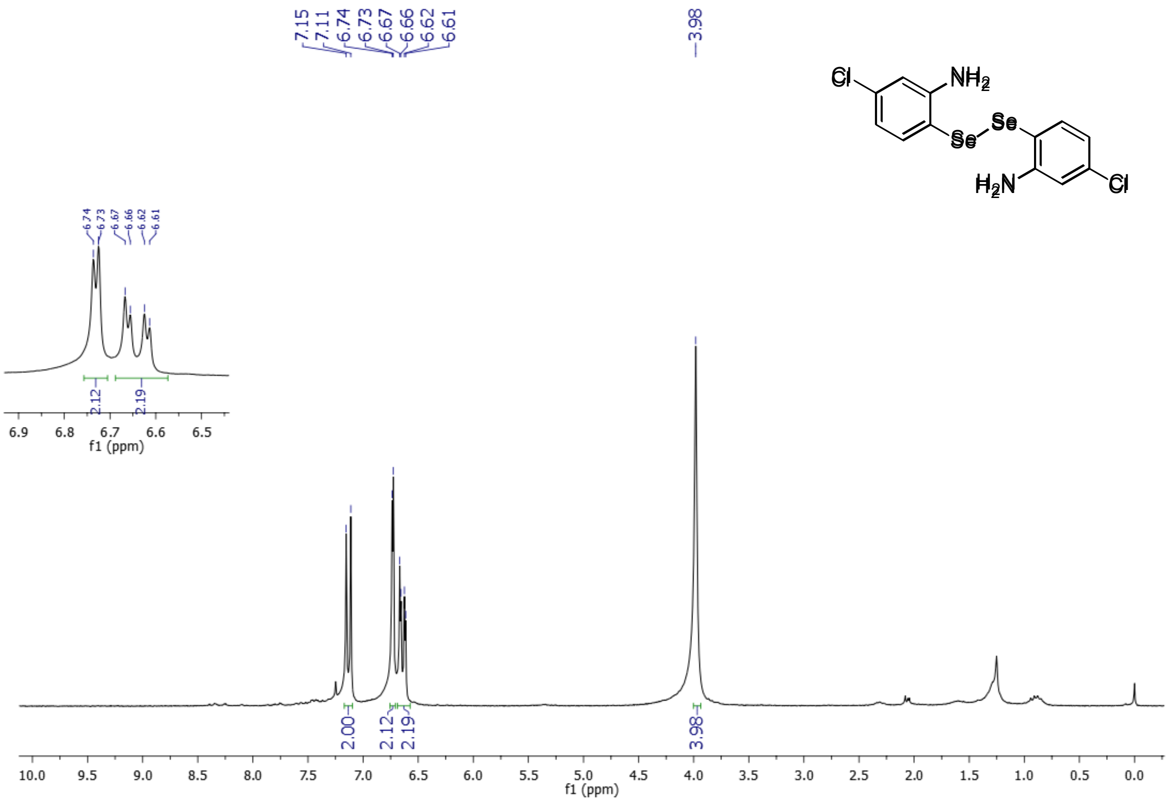

Figure S27.^{1}H NMR (200 MHz, $CDCl_3$) Spectrum of compound **3e**.

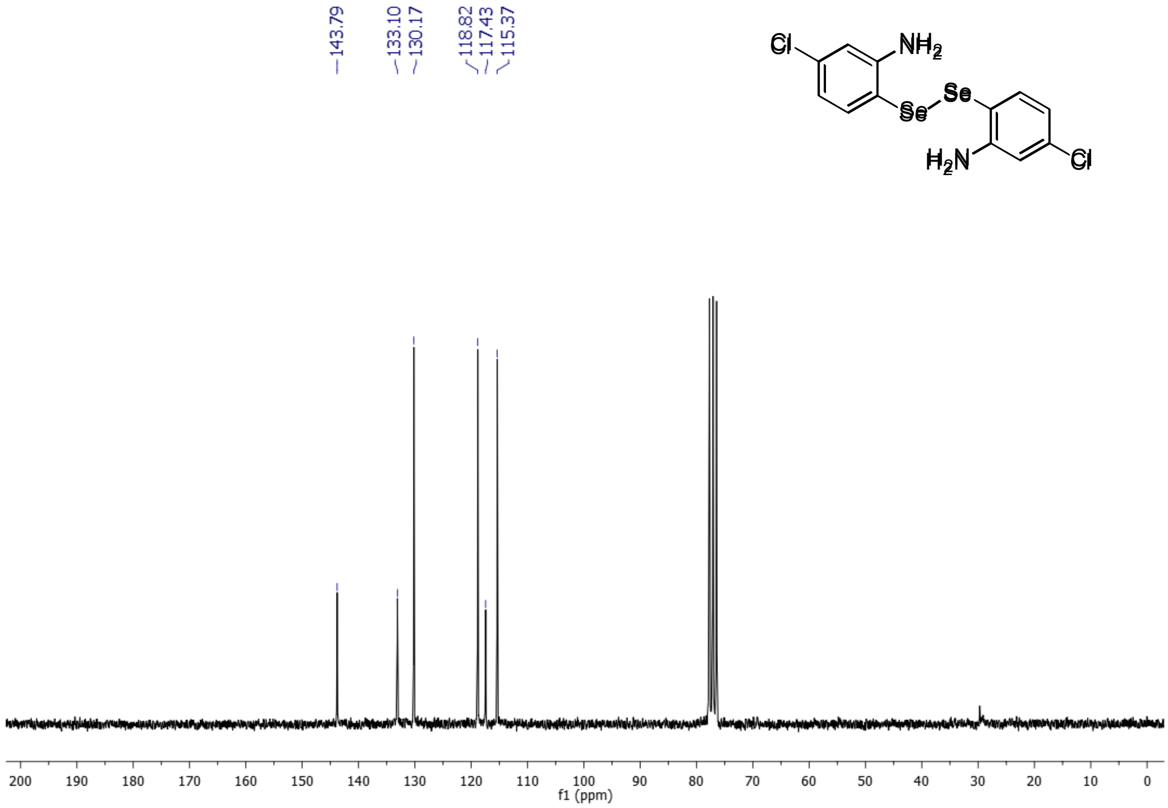

Figure S28.^{13}C NMR (50 MHz, $CDCl_3$) Spectrum of compound **3e**.

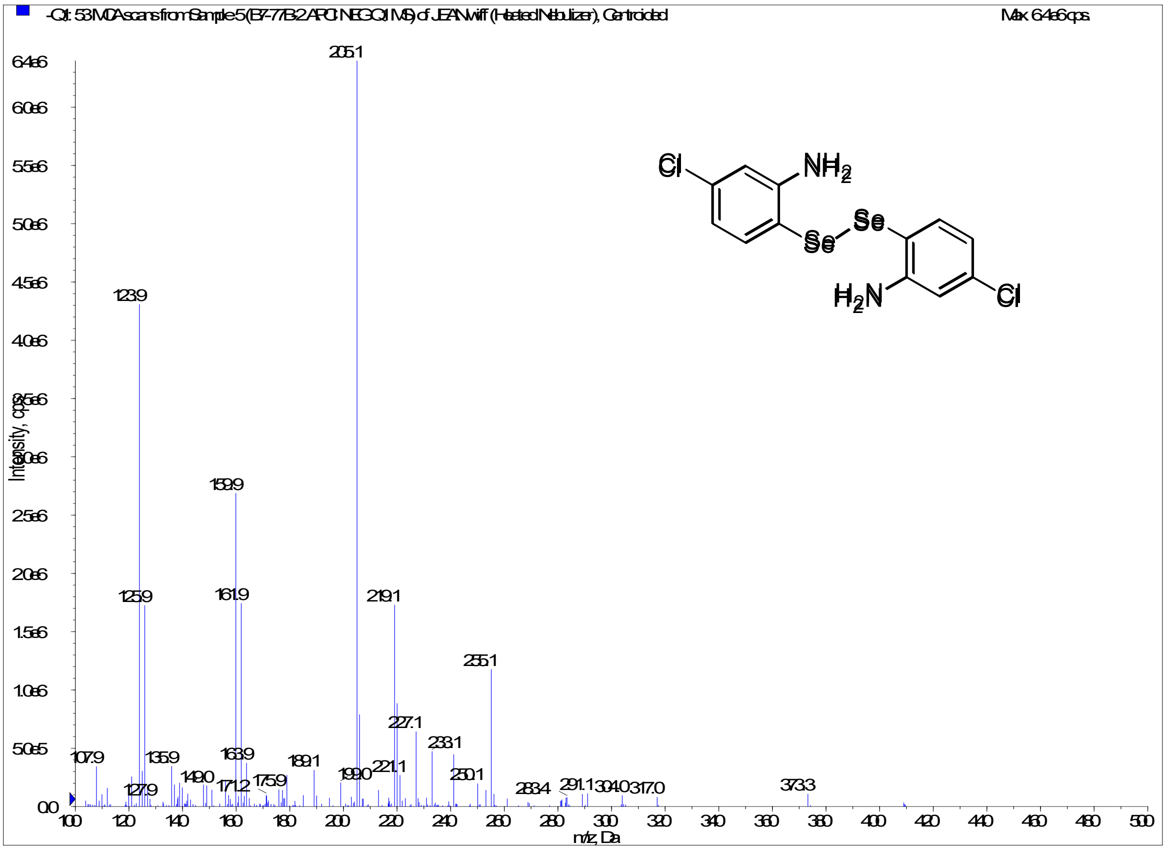

Figure S29. ESI-MS spectrum of compound **3e**.

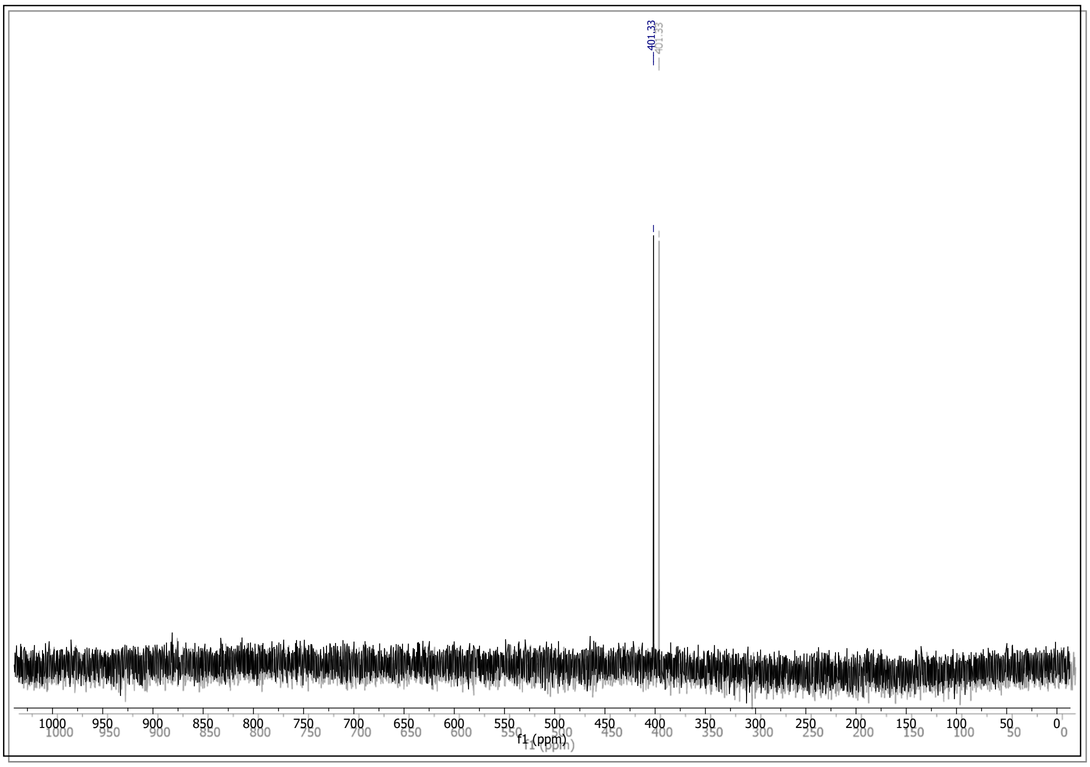

Figure S30. ^{77}Se NMR (76 MHz, $CDCl_3$) Spectrum of compound **3b**.

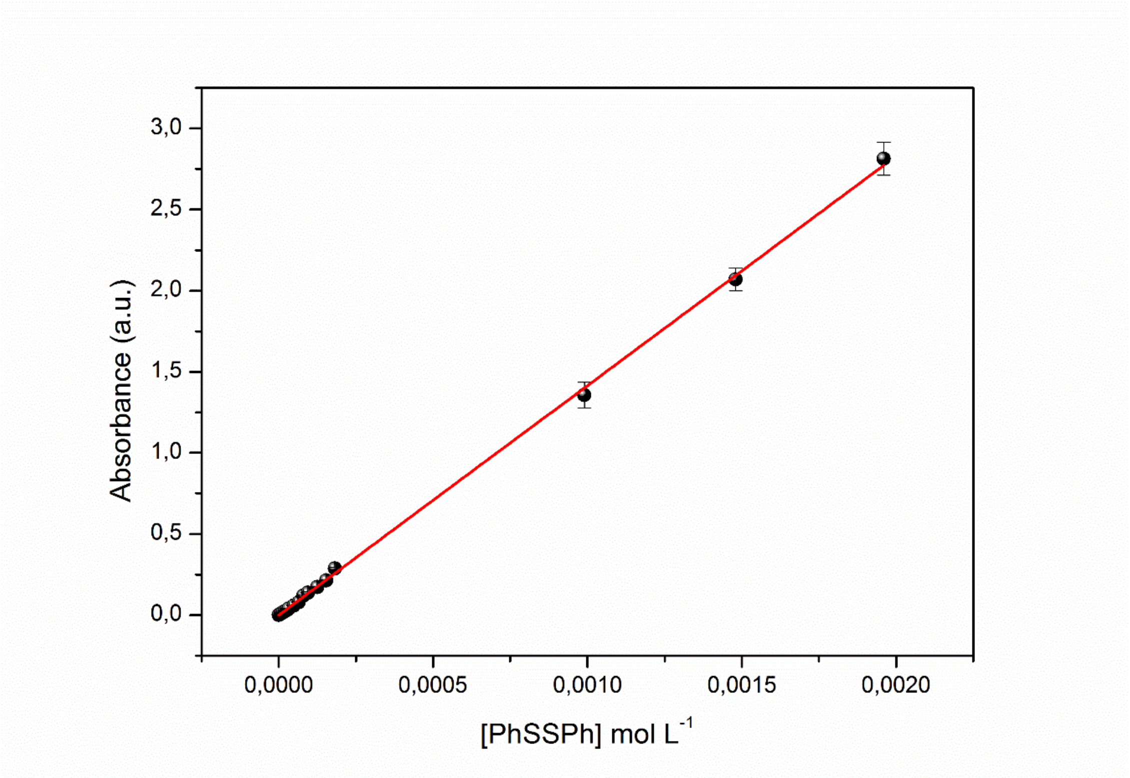

Figure S31. Absorbance plotted against diphenyl disulfide concentration. The red line represents the linear fit. The coefficient of molar absorptivity in 305 nm was 1415 L mol^{-1} cm^{-1} (R^2= 0.9996).

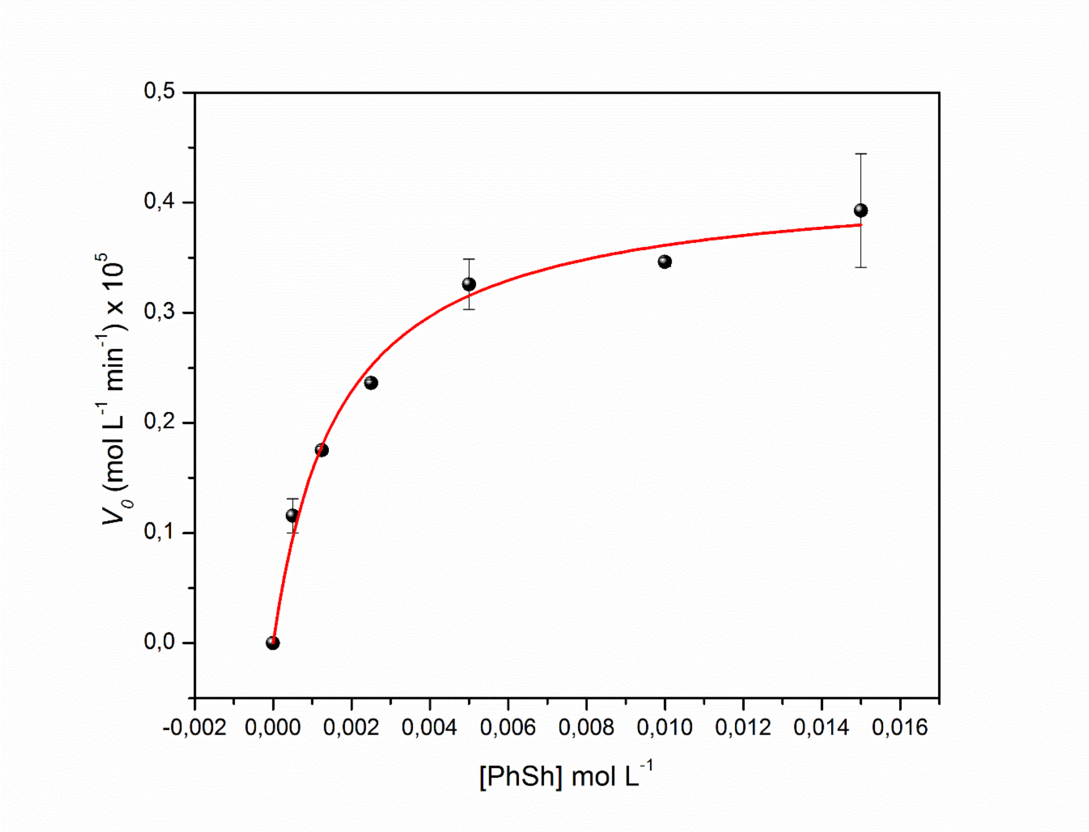

Figure S32. Initial rate (*V_0*) plotted against substrate concentration. The initial rates were calculated from at least two experiments for each concentration of PhSH. The concentrations of ebselen and H_2O_2 were fixed at 5 x 10^{-5} and 15 x 10^{-3} mol L^{-1}, respectively. The red line represents the Michaelis-Menten fit.

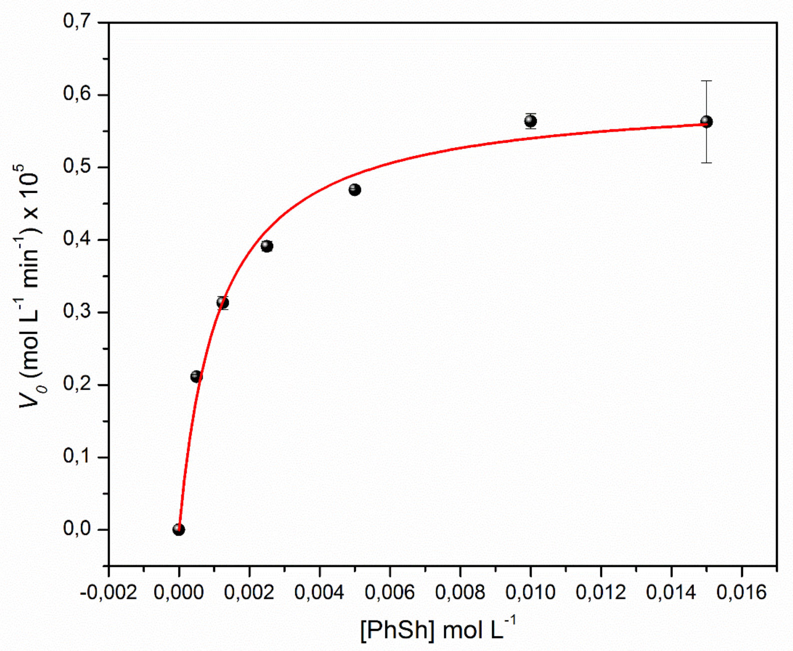

Figure S33. Initial rate (*V_0*) plotted against substrate concentration. The initial rates were calculated from at least two experiments for each concentration of PhSH. The concentrations of diphenyl disulfide and H_2O_2 were fixed at 5 x 10^{-5} and 15 x 10^{-3} mol L^{-1}, respectively. The red line represents the Michaelis-Menten fit.

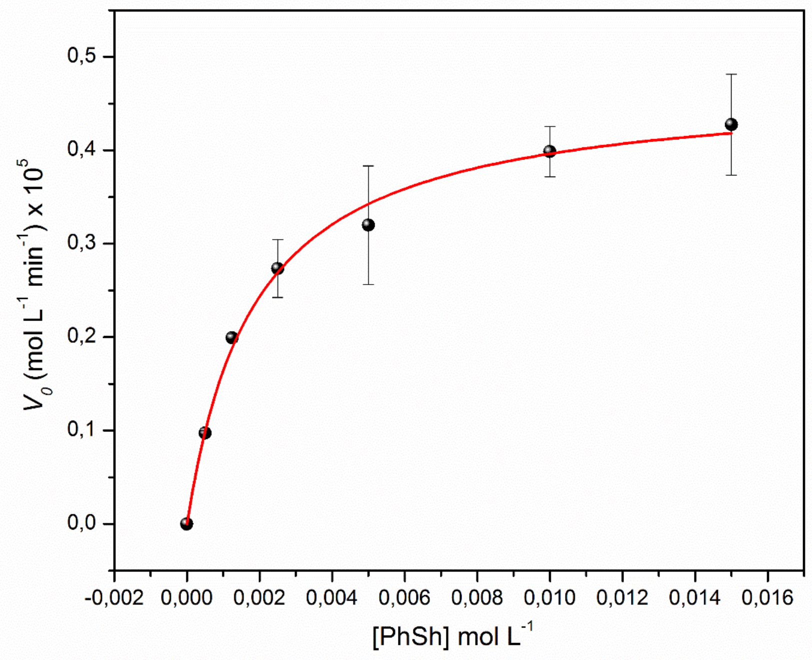

Figure S34. Initial rate (*V_0*) plotted against substrate concentration. The initial rates were calculated from at least two experiments for each concentration of PhSH. The concentrations of **3c** and H_2O_2 were fixed at 5 x 10^{-5} and 15 x 10^{-3} mol L^{-1}, respectively. The red line represents the Michaelis-Menten fit.

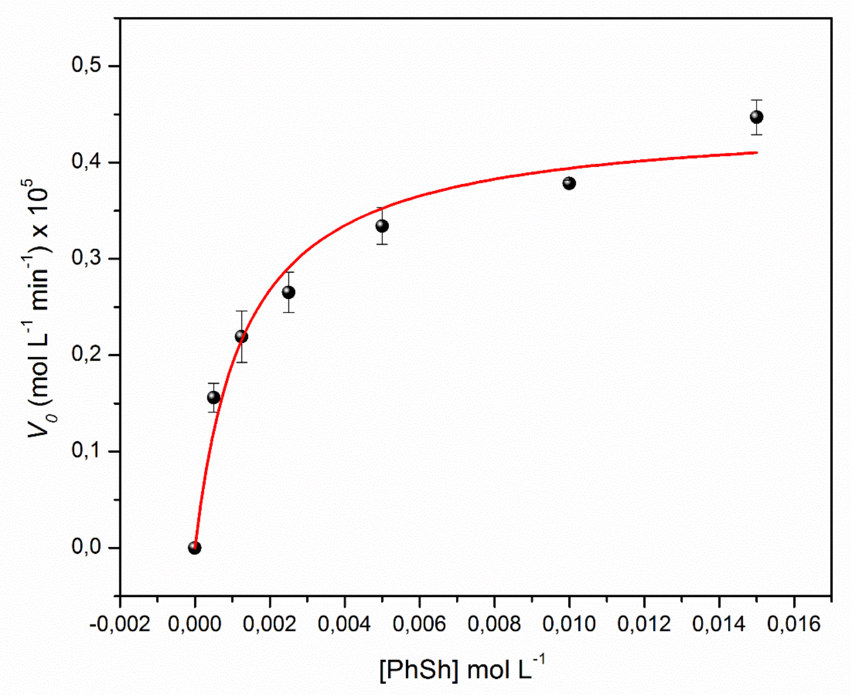

Figure S35. Initial rate (V_0) plotted against substrate concentration. The initial rates were calculated from at least two experiments for each concentration of PhSH. The concentrations of **3a** and H_2O_2 were fixed at 5 x 10^{-5} and 15 x 10^{-3} mol L^{-1}, respectively. The red line represents the Michaelis-Menten fit.

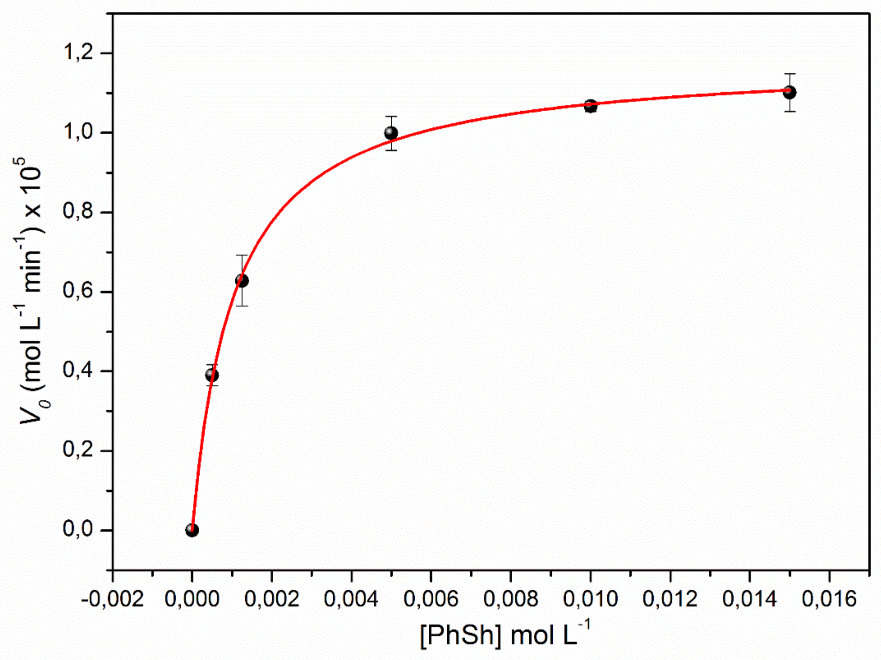

Figure S36. Initial rate (V_0) plotted against substrate concentration. The initial rates were calculated from at least two experiments for each concentration of PhSH. The concentrations of **3b** and H_2O_2 were fixed at 5 x 10^{-5} and 15 x 10^{-3} mol L^{-1}, respectively. The red line represents the Michaelis-Menten fit.

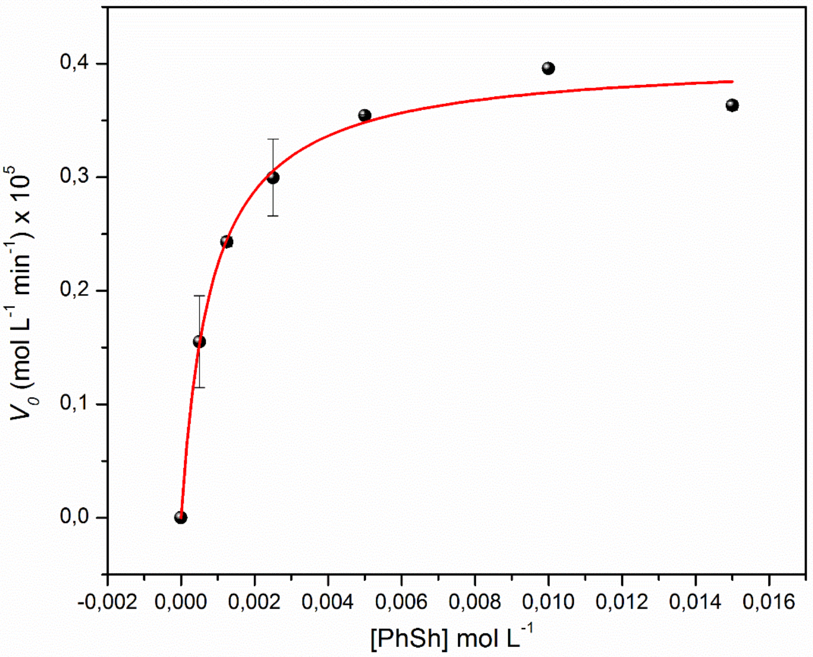

Figure S37. Initial rate (V_0) plotted against substrate concentration. The initial rates were calculated from at least two experiments for each concentration of PhSH. The concentrations of **3d** and H_2O_2 were fixed at 5 x 10^{-5} and 15 x 10^{-3} mol L^{-1}, respectively. The red line represents the Michaelis-Menten fit.

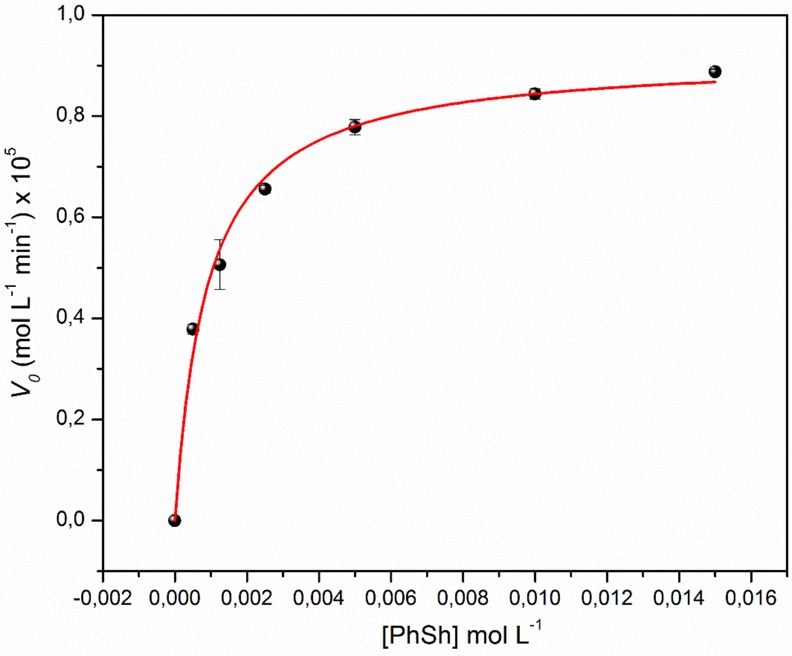

Figure S38. Initial rate (V_0) plotted against substrate concentration. The initial rates were calculated from at least two experiments for each concentration of PhSH. The concentrations of **3e** and H_2O_2 were fixed at 5 x 10^{-5} and 15 x 10^{-3} mol L^{-1}, respectively. The red line represents the Michaelis-Menten fit.

Table S1. Atomic charges of nitrogen and selenium according to NPA.

Aniline-diselenide species		
Group	q_N	q_{Se}
H	-0.772	0.108
Cl	-0.756	0.102
Br	-0.770	0.097
OMe	-0.779	0.063
CF_3	-0.753	0.123
Selenolate species		
Group	q_{Se}	
H	-0.523	
Cl	-0.501	
Br	-0.496	
OMe	-0.549	
CF_3	-0.461	

Table S2. Donor-acceptor second order perturbation analysis.

Aniline-diselenide species		
Group	orbitals	$E^{(2)}$
H	lp(Se) → σ*(NH)	0.81/0.78
Cl		0.75/0.67
Br		0.72/0.67
OMe		0.78/0.79
CF_3		0.57/0.53
Selenolate species		
Group	orbitals	$E^{(2)}$
H	lp(Se) → σ*(NH)	8.61
Cl		8.84
Br		9.05
OMe		8.59
CF_3		8.38

Table S3. DFT-computed energies for optimized geometries (in ha).

	Aniline-diselenide species		
Group	E	G	dispersion correction
H	-5467.165322	-5466.98440	-0.077360
Cl	-6391.014966	-6390.871822	-0.092473
Br	-10715.406914	-10715.266309	-0.096977
OMe	-5696.551147	-5696.328174	-0.099039
CF_3	-6142.481415	-6052.258025	-0.095366
	Selenolate species		
Group	E		E(solv)
H	-2734.167058	-2734.084209	-0.028776
Cl	-3196.089018	-3196.017883	-0.032597
Br	-5358.284719	-5358.215148	-0.033721
OMe	-2848.855074	-2848.744559	-0.035154
CF_3	-3071.825184	-3071.744844	-0.035100

DFT-Optimized coordinates

3a

Lowest vibrations

0: 0.00 cm**-1
1: 0.00 cm**-1
2: 0.00 cm**-1
3: 0.00 cm**-1
4: 0.00 cm**-1
5: 0.00 cm**-1
6: 25.40 cm**-1
7: 44.18 cm**-1
8: 59.89 cm**-1
9: 66.49 cm**-1

H	-1.11995123171601	0.68290730604730	1.09318888943208
C	-0.31678747464897	0.50159292524914	1.81116122526089
C	1.71676592277174	0.05195428086503	3.66891810018365
C	-0.26838230725007	-0.74821576147558	2.47168832716922
C	0.62096754871581	1.49236584038847	2.07651297567091
C	1.65196113586393	1.27651822224948	3.00637841847999
C	0.77364629402148	-0.95513376591016	3.41487682888642
H	0.54644560846461	2.44972182557411	1.55752494287594
H	2.38848393419269	2.05401474989847	3.21295200804860
H	2.49576100684962	-0.13212874647668	4.41056797869604
N	-1.18564704283468	-1.73471077038928	2.15167389997383
H	-1.26237890817569	-2.47658065468711	2.84904758892737
H	-2.09355602705203	-1.39445135674383	1.84197289199333
Se	0.81301340756633	-2.58268482381411	4.45021804706206
Se	-0.41566666533828	-1.72638141244934	6.36319969416012
C	-2.00686391063788	-1.21505473472314	5.41165809483272
C	-4.23181263903806	-0.41059450880111	3.93198858975477
C	-2.13074201802973	0.11574092030916	4.98728262670319
C	-2.99422938105252	-2.18002186363118	5.07089802393036
C	-4.11746236898284	-1.73891942877572	4.33661769710581
C	-3.24256028863571	0.53020345233482	4.25329030396330
H	-1.33681787089179	0.82117054253421	5.23449958753021
H	-4.88764366315297	-2.46492773529972	4.06763111217761
H	-3.32782218316214	1.56502841016932	3.92178122195720
H	-5.10679537314913	-0.10558307585242	3.35490805488276
N	-2.82804881586087	-3.51825972305358	5.36209147973068
H	-2.14265269593607	-3.73347506570535	6.08609423840574
H	-3.66922399290075	-4.08809504783118	5.39337715220523

3b

Lowest vibrations:

0: 0.00 cm**-1
1: 0.00 cm**-1
2: 0.00 cm**-1
3: 0.00 cm**-1

4: 0.00 cm**-1
5: 0.00 cm**-1
6: 19.80 cm**-1
7: 28.23 cm**-1
8: 31.04 cm**-1
9: 47.04 cm**-1

H	4.79743867513424	2.74120186277606	0.41297968375714
C	4.05920423270670	2.90991714602625	-0.37249979036204
C	2.13316450785802	3.29045416196159	-2.35794032006068
C	4.30551847141409	2.39026992415407	-1.66195450071264
C	2.87570156181610	3.58941748573120	-0.09608013615801
C	1.90320799465386	3.80177535754421	-1.08395970935988
C	3.31837455759609	2.60920871688873	-2.66465747858666
H	0.97398250886091	4.31984011948069	-0.85585256306564
H	1.37597977936551	3.40676260402541	-3.13337769601455
N	5.46950201569576	1.71782524249113	-1.92783625666608
H	5.48787245047932	1.14693052339060	-2.77031496873330
H	5.99450018462427	1.34947868711661	-1.14057042856000
Se	3.48299106133143	1.82980116040769	-4.40835571677536
Se	2.94504517415263	-0.47684324271634	-3.69801721635214
C	1.61871041380553	0.01881389151636	-2.40782720293940
C	-0.16749631647462	0.89513904988180	-0.45982407845308
C	1.98141832459906	0.00926397252077	-1.05493453106130
C	0.33964274356270	0.50012867082050	-2.80970218935107
C	-0.54927842035956	0.92497402723761	-1.79860698845202
C	1.09467844441306	0.42747908694295	-0.06627998840500
H	2.98116893023952	-0.32563965660046	-0.78020316849073
H	-1.53043312165904	1.30516166793161	-2.08581731662843
H	1.39029280280264	0.42058007790935	0.98084881851617
N	-0.04059877328525	0.53147345268519	-4.12460665679107
H	0.69613468407031	0.45940754025182	-4.82298113380483
H	-0.82948426546722	1.11737421909345	-4.37910896600160
C	-1.16232446378991	1.34913454597804	0.57314823635719
C	2.66580245571383	4.13479616087899	1.28962884231232
F	-0.59979611608059	1.56558345098889	1.79452089390986
F	-1.79204886938204	2.51203117711008	0.21686211059663
F	-2.16150539449478	0.42343658387770	0.76516507302339
F	1.35202203788763	4.35851198856633	1.57516340927720
F	3.14478694117015	3.29845270125524	2.26070941898640
F	3.30982478703965	5.33685764187590	1.47328251504929

3c

Lowest vibrations:

0: 0.00 cm**-1
1: 0.00 cm**-1
2: 0.00 cm**-1
3: 0.00 cm**-1
4: 0.00 cm**-1
5: 0.00 cm**-1

6:	29.11 cm**-1		
7:	41.05 cm**-1		
8:	49.22 cm**-1		
9:	60.13 cm**-1		

H	4.15604299727505	-0.64350800153578	-1.87636343004221
C	3.77600195109139	-0.92870182405496	-2.85628416415989
C	2.75419150933566	-1.64020163861507	-5.35628554054054
C	3.26591823368876	0.08161637160312	-3.69794361618006
C	3.74481696418546	-2.26451599505266	-3.25290249611268
C	3.26230942082604	-2.62797657411104	-4.52895920188453
C	2.73476034483649	-0.28753669900605	-4.96537872429954
H	3.25454588443421	-3.67773720860038	-4.82268355374111
H	2.32953593066759	-1.91123985052086	-6.32344102354028
N	3.34889836200538	1.40405714768714	-3.29762702518812
H	2.65557331940914	2.01368994580631	-3.73816200923333
H	3.38679633608427	1.53724086375024	-2.28917656990786
Se	1.80220540162292	0.99505892641277	-6.02172280843297
Se	-0.51168200283967	0.42385071536901	-5.18374212841024
C	-0.00095719987762	-0.10595800246297	-3.42998645778271
C	0.92966138697260	-0.95388026935196	-0.92643880981784
C	0.18644947665843	-1.47396624822236	-3.15457578524457
C	0.29955146509508	0.85729655686325	-2.42620400707857
C	0.75249026619016	0.40954057022529	-1.16533443012687
C	0.62628933479078	-1.91120389795268	-1.91749216212599
H	-0.01531832908301	-2.19803379185336	-3.94372542447860
H	0.99903903269320	1.15194963569031	-0.40717423783104
H	0.79120682867211	-2.96859300416380	-1.71178896935327
O	1.40982980720316	-1.46143332674273	0.24981097626862
C	1.80628890710427	-0.52503260361336	1.26964598213953
H	2.60758362651894	0.13611578358194	0.90495354938058
H	2.17803290365527	-1.13442163686128	2.09920260202398
H	0.95015029189037	0.07928286649099	1.60565385002682
N	0.24150096351328	2.20286134916555	-2.69659370974379
H	-0.28688691153990	2.46923965561430	-3.52733357634196
H	0.17507560863827	2.84208979658086	-1.90992828098049
O	4.15826656813786	-3.29979292894537	-2.46128419830030
C	4.51192371601629	-2.99187921751813	-1.09797644749597
H	5.38956850896804	-2.32898921309123	-1.05609111008641
H	4.75587530468394	-3.95298616330849	-0.63483909537002
H	3.66325379047574	-2.52735208925656	-0.57270796600777

3d

Lowest vibrations

0:	0.00 cm**-1
1:	0.00 cm**-1
2:	0.00 cm**-1
3:	0.00 cm**-1
4:	0.00 cm**-1
5:	0.00 cm**-1

6: 21.29 cm**-1
7: 30.06 cm**-1
8: 35.53 cm**-1
9: 59.10 cm**-1

H	1.72741878838432	3.36675068476418	-0.54749410379097
C	2.02552767006022	3.02549041934578	-1.53842963667332
C	2.79842642110032	2.07701005295066	-4.05211386563797
C	1.07648726380430	3.02034990301711	-2.58632136173693
C	3.30735365154790	2.55527487864684	-1.77096452413821
C	3.73440440799733	2.09153784605571	-3.01994891625763
C	1.48225622243754	2.51700448257266	-3.85480120480051
H	4.74875101418722	1.72945047182429	-3.17575461719490
H	3.08265797823365	1.68186840391415	-5.02802747083180
N	-0.18524270902245	3.53257341834246	-2.36971623141844
H	-0.90582661814027	3.16112365195404	-2.99373825970885
H	-0.48231728391131	3.56149517395526	-1.39719826899381
Se	0.17209690701440	2.24726234887191	-5.22837580352926
Se	-0.14124908409351	-0.18705434217868	-4.81681627276899
C	0.13371731394446	-0.12385930081514	-2.92630945030130
C	0.70041185549990	0.13956813965627	-0.22915036166525
C	1.38940091819808	-0.49212417293927	-2.42192081800853
C	-0.86881375661683	0.38814217667867	-2.05454963615824
C	-0.55912072771877	0.50695471585638	-0.67931047651289
C	1.69173258737413	-0.38025648591162	-1.06754809153788
H	2.14906943553830	-0.85716343795437	-3.11247578460593
H	-1.30148353794476	0.91891594725214	0.00418829194740
H	2.67513842002545	-0.64764718091621	-0.68744600280413
Br	1.12117173653266	0.40376708007304	1.63268866924783
N	-2.06540995599422	0.85263863944408	-2.53634205738654
H	-2.32306422615339	0.54961216569945	-3.47515008172795
H	-2.83476406810794	0.95464607453254	-1.88111731994186
Br	4.53836937582325	2.49101824530766	-0.28999634306314

3e

Lowest vibrations:
0: 0.00 cm**-1
1: 0.00 cm**-1
2: 0.00 cm**-1
3: 0.00 cm**-1
4: 0.00 cm**-1
5: 0.00 cm**-1
6: 21.26 cm**-1
7: 36.23 cm**-1
8: 43.40 cm**-1
9: 60.25 cm**-1

H	0.88697127019777	4.11295775029334	-0.34889181233324
C	1.83365811626601	3.71331311240774	-0.71146468529065
C	4.25714696744385	2.63259849411581	-1.59112080310255

C	1.96343559663932	3.34820933891733	-2.07056168971290
C	2.89778318852689	3.52603937564369	0.15539667671070
C	4.12852177232382	3.00042558407069	-0.25359122189582
C	3.20006103516693	2.78687619604530	-2.49813116356264
H	4.94694310161100	2.86512083443131	0.45111770997984
H	5.18908154447761	2.18481223926003	-1.93764768339732
N	0.92433171313051	3.58353448079605	-2.94476733193137
H	0.92449595657326	2.98849224024976	-3.77676218963768
H	0.00506370722313	3.66965926457227	-2.51807762208665
Se	3.34418141002663	2.05631802824798	-4.26602869719739
Se	2.97137445121165	-0.30883956130604	-3.60420678160435
C	1.73273070362307	0.05364025344775	-2.19426614589297
C	0.05968658841637	0.74937436669295	-0.09898523758325
C	2.20222405627452	0.04530737519793	-0.87300253674956
C	0.38796886415530	0.42656178807230	-2.47496341678784
C	-0.45022689185271	0.76716680822322	-1.38873836169969
C	1.37596579012868	0.37994456886357	0.19574904993954
H	3.24302091796439	-0.21625243191635	-0.68501274993986
H	-1.47904409818880	1.07442106523349	-1.57659344780222
H	1.75020137269933	0.39040889333436	1.21743539664993
Cl	-0.99837845973744	1.24812250747542	1.23126963533667
N	-0.06378338155135	0.55380307811011	-3.76273705665362
H	0.49082959453543	0.09666030570070	-4.48563204822562
H	-1.06754930932086	0.56267574370305	-3.91846289118708
Cl	2.67441442203567	3.93896830011627	1.86403710565757

Selenolate a
Lowest vibrations:

0: 0.00 cm**-1
1: 0.00 cm**-1
2: 0.00 cm**-1
3: 0.00 cm**-1
4: 0.00 cm**-1
5: 0.00 cm**-1
6: -148.56 cm**-1 ***imaginary mode***
7: 125.86 cm**-1
8: 208.41 cm**-1
9: 226.12 cm**-1

H	-1.98379784213075	0.86108704767195	-0.15521264293190
C	-1.10826827158794	0.48053001853928	-0.68481847290146
C	1.11326193750329	-0.48329548815015	-2.09501041601034
C	-1.08314022429435	0.47026564670907	-2.07569338840646
C	0.00084500748212	-0.00129275021577	0.01674928294306
C	1.10735065764388	-0.48153116172805	-0.69913092023730
C	0.00921409455851	-0.00328665638315	-2.83202073912128
H	1.97892957693919	-0.85974946580347	-0.16120250927580
H	1.98355268341027	-0.86070287421280	-2.63382293232877
N	-2.23708893048175	0.97086865065798	-2.83872476126260

H	-1.94926718440342	0.86746778823246	-3.86190305717725
H	-3.09202561101629	0.42273176961366	-2.67804639918635
H	-2.44754899407057	1.95855247564631	-2.64704935800886
Se	-0.05324676276851	0.02743312906847	-4.75082259878933
H	-0.00018013678368	-0.00090812964579	1.10721891269463

Selenolate b

Lowest vibrations:
0: 0.00 cm**-1
1: 0.00 cm**-1
2: 0.00 cm**-1
3: 0.00 cm**-1
4: 0.00 cm**-1
5: 0.00 cm**-1
6: -167.34 cm**-1 ***imaginary mode***
7: 1.62 cm**-1
8: 65.94 cm**-1
9: 141.98 cm**-1

H	-2.01155895040243	0.83164680446368	-0.16198957568798
C	-1.12924605782821	0.46737404074564	-0.68829897901653
C	1.11909879474808	-0.46419277606597	-2.08318559205302
C	-1.09096497370755	0.46535035058600	-2.07441361980188
C	-0.01836188937624	-0.01348453352356	0.01373057023330
C	1.10689691013610	-0.47438677350269	-0.69393052920416
C	0.01389926840768	0.00933429601528	-2.82860969820299
H	1.97665378243913	-0.84818094895431	-0.15203689891839
H	1.99779122265613	-0.82867307436704	-2.61573132168029
N	-2.24493387792364	0.95613093501172	-2.83946320300421
H	-1.94856716768417	0.86958354428693	-3.86130131530105
H	-3.09188412359622	0.39124684148297	-2.69378199027972
H	-2.47327862104435	1.93801516512940	-2.63772996837933
Se	-0.03088871100356	0.05075188969799	-4.73301719319549
C	0.00669340823619	0.00354593482474	1.50897073007007
F	-1.22587209777372	0.20028564674805	2.06121018443481
F	0.49466644121173	-1.16460816272538	2.03623198830560
F	0.81614664250504	1.00019082014656	2.01081641168124

Selenolate c

Lowest vibrations:
0: 0.00 cm**-1
1: 0.00 cm**-1
2: 0.00 cm**-1
3: 0.00 cm**-1
4: 0.00 cm**-1
5: 0.00 cm**-1
6: -143.85 cm**-1 ***imaginary mode***
7: 65.76 cm**-1
8: 118.16 cm**-1
9: 172.83 cm**-1

H	-1.50681690957051	1.37048059098675	-0.13540902957073
C	-0.76962617629341	0.78707796371293	-0.68522952241761
C	1.07139479557236	-0.70835639935190	-2.17993832300391
C	-0.93561679036207	0.56943964997234	-2.05381243235639
C	0.35486392763825	0.23769929184576	-0.05716480984745
C	1.27186678844256	-0.51202876021834	-0.81801545664600
C	-0.04894250775574	-0.17015360626903	-2.85195281294989
H	2.14560507057469	-0.93754279669828	-0.32176555155499
H	1.79778227261921	-1.29275940332742	-2.74644973891700
N	-2.10821481603433	1.13765323041494	-2.73648420179382
H	-2.00006180542523	0.83150542036525	-3.75414549228536
H	-2.99983867992893	0.77839830769301	-2.37168415099993
H	-2.13540253290684	2.16511822758803	-2.69829962334319
Se	-0.38271295777702	-0.40605859738147	-4.73324083666604
O	0.64065130978495	0.37593854634706	1.27798654079079
C	-0.28083480528555	1.14723760239267	2.06869385217076
H	-0.34929413550508	2.18292561729720	1.70010406981074
H	0.12750726016421	1.14387919135822	3.08437259219039
H	-1.28165930795150	0.68736592327226	2.06809492738969

Selenolate d

Lowest vibrations:

0: 0.00 cm**-1
1: 0.00 cm**-1
2: 0.00 cm**-1
3: 0.00 cm**-1
4: 0.00 cm**-1
5: 0.00 cm**-1
6: -144.78 cm**-1 ***imaginary mode***
7: 70.38 cm**-1
8: 156.88 cm**-1
9: 163.29 cm**-1

H	0.56257357662590	-2.01445105662372	-1.59170963301840
C	1.20672788182421	-1.14659234573054	-1.73407558380459
C	2.84593001855644	1.10631545090188	-2.07403102680738
C	1.02684316869084	0.00610175962744	-0.97618049537681
C	2.23477612282847	-1.14941280902985	-2.67543382764048
C	3.05584559298310	-0.03191848042703	-2.85323969605365
C	1.82219610348520	1.16477381451323	-1.10463876342506
H	3.85539402566823	-0.04486656064741	-3.59429786803759
H	3.49131603436344	1.97374246499957	-2.21872715968098
N	-0.04557511057684	0.06130416765836	0.02541214191152
H	0.04904306658868	1.03422853069413	0.46082974055114
H	-0.98378931859589	-0.02860049796480	-0.38597401520395
H	0.05334086346586	-0.64888148107665	0.76258236000127
Se	1.48787524889724	2.69408387449993	-0.00287965888474
Br	2.52193272519511	-2.72406683139454	-3.74675651453027

Selenolate e

Lowest vibrations:

0: 0.00 cm**-1
1: 0.00 cm**-1
2: 0.00 cm**-1
3: 0.00 cm**-1
4: 0.00 cm**-1
5: 0.00 cm**-1
6: -147.20 cm**-1 ***imaginary mode***
7: 80.84 cm**-1
8: 171.54 cm**-1
9: 184.86 cm**-1

H	-1.89635377961877	1.04776911583318	-0.15742277193477
C	-1.07015715519284	0.57747319876514	-0.69092447408634
C	1.04353984236833	-0.62842734272926	-2.08735486264903
C	-1.07872470742160	0.47544280925358	-2.07801474169380
C	0.02915558819733	0.05879593318371	-0.00847154377253
C	1.08684873335554	-0.54412500765005	-0.69581441669007
C	-0.04453205566667	-0.12102319902659	-2.82931683893426
H	1.94060534272698	-0.94437475856781	-0.14898779598864
H	1.87324890687792	-1.09924731614356	-2.61573711339363
N	-2.21570871751203	1.00746506402498	-2.84018739047076
H	-1.96935504893128	0.80659084954999	-3.86139736580662
H	-3.10447436866507	0.54218747821296	-2.61383436633698
H	-2.34228304579858	2.02079470788395	-2.71899432818240
Se	-0.16221428239861	-0.20933002510357	-4.73940807730742
Cl	0.07905474767938	0.16871849251335	1.75818608724727

9. References

1. (a) Rheinboldt, H.; Giesbrecht, E. *J. Am. Chem. Soc.* **1950**, *72*, 866; (b) Syper, L.; Młochowski, J. *Tetrahedron* **1988**, *44*, 6119.
2. Giurg, M.; Kowal, E.; Muchalski, H.; Syper, L.; Młochowski, J. *Synth. Commun.* **2009**, *39*, 251.
3. (a) Galet, V.; Bernier, J.-L.; Hénichart, J.-P.; Lesieur, D.; Abadie, C.; Rochette, L.; Lindenbaum, A.; Chalas, J.; Faverie, J.-L. R.; Pfeiffer, B.; Renard, P. *J. Med. Chem.* **1994**, *37*, 2903; (b) Kloc, K.; Młochowski, J.; Osajda, K.; Syper, L.; Wójtowicz, H. *Tetrahedron Lett.* **2002**, *43*, 4071.
